# Endogenous virophages are active and mitigate giant virus infection in the marine protist *Cafeteria burkhardae*

**DOI:** 10.1073/pnas.2314606121

**Published:** 2024-03-06

**Authors:** Anna Koslová, Thomas Hackl, Felix Bade, Alexander Sanchez Kasikovic, Karina Barenhoff, Fiona Schimm, Ulrike Mersdorf, Matthias G. Fischer

**Affiliations:** ^a^Department of Biomolecular Mechanisms, Max Planck Institute for Medical Research, Heidelberg 69120, Germany; ^b^Faculty of Science and Engineering, Groningen Institute for Evolutionary Life Sciences, University of Groningen, Groningen 9747 AG, The Netherlands

**Keywords:** virophage, giant virus, protist, antiviral defense, microbial ecology

## Abstract

Endogenous viral elements (EVEs) are common genetic passengers in various protists. Some EVEs represent viral fossils, whereas others are still active. The marine heterotrophic flagellate *Cafeteria burkhardae* contains several EVE types related to the virophage mavirus, a small DNA virus that parasitizes the lytic giant virus CroV. We hypothesized that endogenous virophages may act as an antiviral defense system in protists, but no protective effect of virophages in wild host populations has been shown so far. Here, we tested the activity of virophage EVEs and studied their impact on giant virus replication. We found that endogenous mavirus-like elements (EMALEs) from globally distributed *Cafeteria* populations produced infectious virus particles specifically in response to CroV infection. However, reactivation was stochastic, often inefficient, and poorly reproducible. Interestingly, only one of eight EMALE types responded to CroV infection, implying that other EMALEs may be linked to different giant viruses. We isolated and cloned several reactivated virophages and characterized their particles, genomes, and infection dynamics. All tested virophages inhibited the production of CroV during coinfection, thereby preventing lysis of the host cultures in a dose-dependent manner. Comparative genomics of different *C. burkhardae* strains revealed that inducible EMALEs are common and are not linked to specific geographic locations. We demonstrate that naturally occurring virophage EVEs reactivate upon giant virus infection, thus providing a striking example that eukaryotic EVEs can become active under specific conditions. Moreover, our results support the hypothesis that virophages can act as an adaptive antiviral defense system in protists.

## Significance

Single-celled eukaryotes are parasitized by viruses just like other life forms, but compared to bacteria or animals, their defense systems against viral infection remain unknown. Virophages could be one such mechanism since these small DNA viruses often inhibit giant DNA viruses while leaving their protist hosts unharmed. In this study, we demonstrate that certain virophage genomes that are integrated in wild populations of the marine protist *Cafeteria burkhardae* become active upon infection with the giant virus CroV and are able to protect host populations from lysis by CroV. We show that this virophage-based defense is giant-virus-specific and is present in protists from around the globe. Our findings strongly suggest that some protists use beneficial viruses to fend off lytic viruses.

Viruses of the class *Maveriviricetes*, commonly known as virophages, are small double-stranded (ds) DNA viruses, which infect unicellular eukaryotic cells but require a coinfecting giant virus for their replication (1, 2). Associated giant viruses belong to the order *Imitervirales* in the phylum *Nucleocytoviricota,* also known as nucleocytoplasmic large DNA viruses (NCLDVs). Virophages propagate in viral factories formed by giant viruses and can interfere with giant virus replication (3–6). While only a few virophages have been isolated in culture, metagenomic studies revealed that these viruses are highly diverse and are found in various ecosystems around the planet (7–9). Virophages probably regulate population dynamics of giant viruses and their eukaryotic hosts and may thus have a considerable influence on ecosystem functioning in protistan communities (10–12).

Virophages overlap in their gene content with Maverick/Polinton elements (MPEs) and Polinton-like viruses (PLVs), and although virophages, MPEs, and PLVs represent phylogenetically distinct groups (1), they probably have a common origin (13, 14). MPEs were initially described as self-synthesizing transposons found in diverse unicellular eukaryotes and animals (15, 16). However, as many MPEs carry viral morphogenesis genes (17), they qualify as endogenous viruses. PLVs were discovered in metagenomes (18) and exhibit a particularly high degree of genetic diversity. Recently, it was shown that PLVs, MPEs, and virophages are much more prevalent in protist genomes that previously assumed (19–23).

These genomic surveys stand in stark contrast to functional studies, and little is known about the lifestyles and eco-evolutionary impacts of these elements. To date, only two PLVs and a handful of virophages have been isolated in culture. Whereas PLVs can either depend on a giant virus (22, 24) or replicate independently in the host cell (25), all virophages isolated so far coreplicate with a specific giant virus (3, 4, 26, 27). The strong correlation between virophage and giant virus gene promoter motifs and the timing of virophage DNA replication suggest that virophages use the late transcription machinery of the giant virus to express their genes (4, 28, 29). Virophage and PLV sequences that are integrated in cellular genomes are usually silent, and although some of their genes can be transcribed (21), so far, no production of virus particles was observed without the involvement of a giant virus.

The dual lifestyle of virophages has experimentally only been shown for mavirus (30). Mavirus replicates during coinfection with the giant Cafeteria roenbergensis virus (CroV); however, it can also integrate into the nuclear genome of *Cafeteria* spp. independently of CroV. Provirophage genes are induced by infection with CroV, which results in the formation of infectious virophage particles. The newly produced mavirus particles inhibit CroV replication in subsequent rounds of infection (5). Genome-integrating virophages may therefore represent a unique example of an inducible antiviral defense system in eukaryotic microbes (5, 31). The virophage defense hypothesis is further supported by the finding that genomes of wild *C. burkhardae* populations contain dozens of copies of endogenous mavirus-like elements (EMALEs) (32). We described eight types of EMALEs based on GC content, nucleotide similarity, and phylogenetic analysis. Different EMALE types also carry distinct promoter motifs implicating interactions with different giant viruses.

In uninfected *Cafeteria* cultures, EMALEs are transcriptionally silent, and no virion formation is observed. Here, we analyzed whether EMALEs from wild flagellate populations are functional and whether they can protect against the lytic giant virus CroV. We demonstrate that EMALEs reactivate in many strains of *C. burkhardae* when infected with CroV. We also show that only EMALEs of one type respond to CroV infection and that reactivated virophages display properties similar to mavirus. Our results provide strong evidence that endogenous virophages from wild protist populations are active, thus corroborating the hypothesis that virophages can act as an adaptive antiviral defense system in marine flagellates. We show that virophage-based defense is an ecologically relevant process in a globally distributed protist species.

## Results

### CroV Infection of *C. burkhardae* Reactivates Endogenous Virophages of EMALE Type 4.

**Fig. 1.**
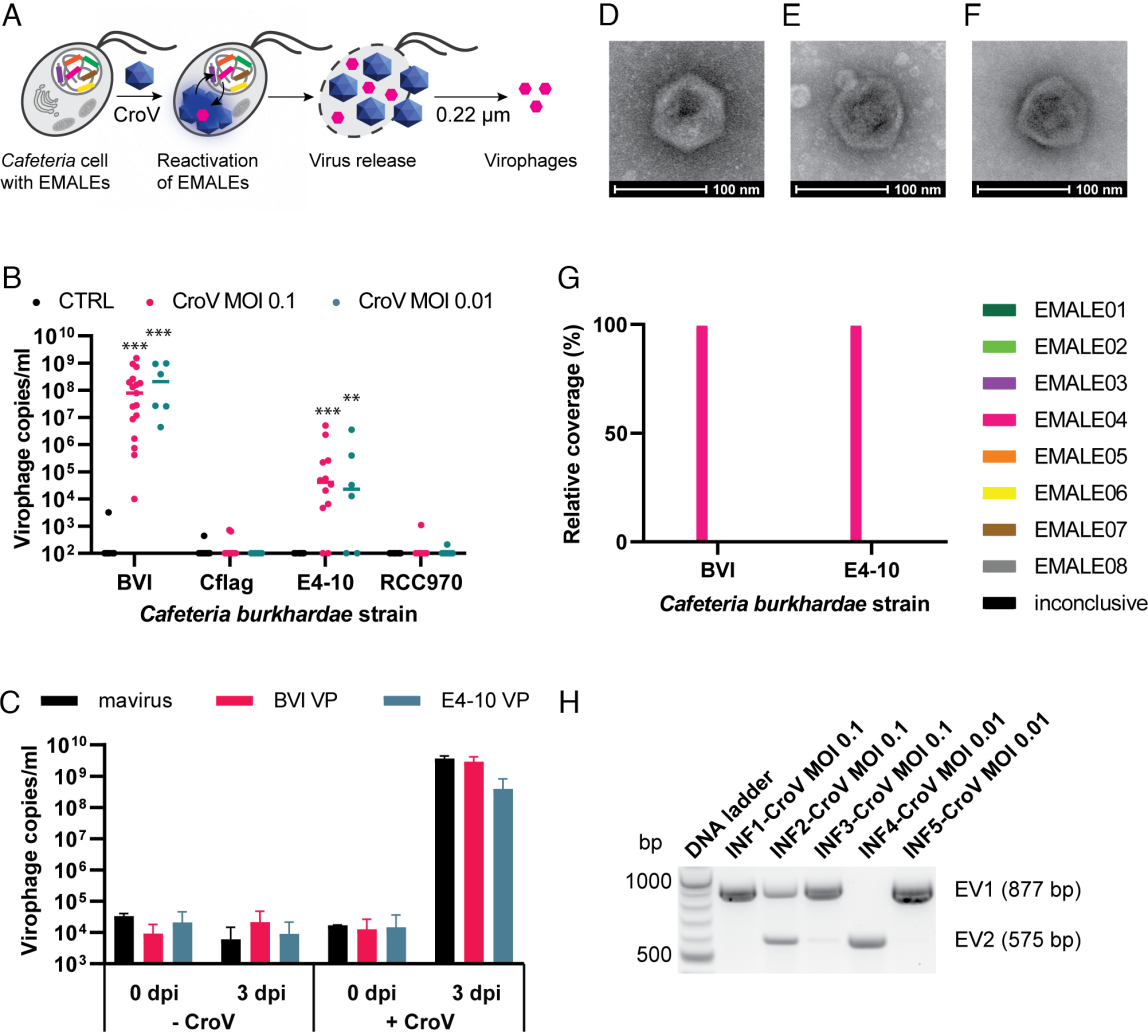
*Cafeteria burkhardae* releases EMALE04 virophages upon CroV infection. (*A*) *C. burkhardae* strains containing various EMALEs (depicted as colored lines) were infected with CroV. After cell lysis, the 0.22-µm filtrates of culture supernatants were analyzed for the presence of virophage DNA and particles by qPCR. (*B*) Quantification of type 4 EMALEs produced during CroV infection of four *C. burkhardae* strains. The 0.22-μm-filtered lysates were treated with DNase prior to DNA extraction and qPCR analysis with EMALE04-specific *polB* primers. Significant differences compared to uninfected controls are marked by asterisks (**P = 0.01 to 0.001, ***P < 0.001). (*C*) Cultures of *C. burkhardae* strain RCC970 were inoculated with virophages (mavirus or BVI- and E4-10-derived virophages) alone or together with CroV. Replication of virophage DNA was analyzed 3 days post infection (dpi) using qPCR with EMALE04-specific primers. (*D*–*F*) Negative stain electron micrographs of mavirus (*D*), BVI VP after reactivation (*E*), and E4-10 VP after reactivation and propagation in host strain RCC970 (*F*). (*G*) Postlysis culture supernatants of CroV-infected strains BVI and E4-10 were 0.22 µm filtered and concentrated, followed by Illumina DNA sequencing. DNA reads were mapped to the respective *C. burkhardae* genome assemblies, and for each host strain, a cumulative coverage for all EMALE types was calculated. (*H*) PCR products specific for reactivated virophages EV1 and EV2 from five individual CroV infections of host strain E4-10.

We tested whether endogenous virophage elements in the marine protist *C. burkhardae* are able to form infectious particles upon infection with the giant virus CroV (Fig. 1*A*). CroV has been shown to induce the virophage mavirus from a provirophage-carrying host strain that was produced in the laboratory (5). We inoculated the *C. burkhardae* strains BVI, Cflag, E4-10, and RCC970 with CroV at multiplicities of infection (MOI) of 0.1 or 0.01. These host strains have fully annotated genomes and contain a variety of EMALEs (32, 33). The infected cell populations died within several days, even though we observed differences in lysis behavior among individual host strains. Cultures of Cflag, E4-10, and RCC970 lysed 3 to 5 d post infection (pi) and permitted high levels of CroV replication (>10^8^ genome copies/mL, *SI Appendix*, Fig. S1 *A*–*C*). In contrast, lysis of strain BVI was delayed to 8 dpi, and CroV replication levels were low (<10^7^ genome copies/mL, *SI Appendix*, Fig. S1*D*). Occasionally, we observed that CroV infection in strain BVI was abortive, as indicated by a lack of viral replication and no cell lysis within 10 d (*SI Appendix*, Fig. S1*E*).

Following lysis, we filtered the culture supernatants through 0.22-µm-pore-size filters to separate virophage capsids (50 to 70 nm in diameter) from cell-associated virophage DNA. The filtrate was treated with DNase to remove unpackaged DNA, followed by DNA extraction and qPCR analysis with primers targeting conserved regions within the retroviral integrase (*rve-Int*) and DNA polymerase B (*polB*) genes of different EMALE types. We first tested for the presence of DNA from EMALEs of type 4 (EMALE04), because members of this clade are the closest relatives to mavirus and contain the late promoter motif of CroV (32). Irrespective of MOI, we found EMALE04-specific DNA in the filtered lysates of strains BVI and E4-10, but not in Cflag or RCC970, nor in uninfected controls (Fig. 1*B* and *SI Appendix*, Fig. S2). Filtrates prepared from CroV-infected BVI cultures contained higher concentrations of EMALE04 DNA than those from strain E4-10 (on average 2.5 × 10^8^ copies/mL versus 6.7 × 10^5^ copies/mL), although the virophage response was faster in E4-10 than in BVI (*SI Appendix*, Fig. S1), and virophage DNA concentrations varied considerably between individual infections. Some CroV-infected E4-10 cultures had undetectable levels of 0.22-µm-filterable EMALE04 DNA, suggesting inefficient or absent reactivation of endogenous virophages. These fluctuations indicate that the reactivation of endogenous virophages is stochastic or partly controlled by factors that may vary between infection experiments. Even within a genetically homogeneous host population, differences in cell cycle and physiological states may greatly influence the reactivation behavior.

To determine the timing of virophage reactivation in CroV-infected strains BVI and E4-10, we quantified virophage DNA daily until lysis. Filterable virophage DNA appeared 2 to 3 dpi and increased over the time, reaching the highest concentrations during lysis (*SI Appendix*, Fig. S1 *C* and *D*).

Genome analysis of *C. burkhardae* revealed only one partial (or partially assembled) copy of EMALE04 in strains Cflag and RCC970. In contrast, there were two partial EMALE04 copies in strain E4-10, and 16 copies (2 fully assembled, 14 partially assembled) in strain BVI (32). The higher reactivation levels of type 4 EMALEs in strain BVI thus correlate with a higher copy number of these elements in the host genome. However, the faster virophage response in host strain E4-10 appears to be correlated with higher levels of CroV replication, compared to strain BVI (*SI Appendix*, Fig. S1). The type 4 EMALEs in Cflag and RCC970 appear inactive, potentially because of truncation.

To confirm that reactivation produced infectious virophage particles, we amplified them in host strain RCC970, which lacks CroV-responsive type 4 EMALEs. We coinoculated RCC970 cells with CroV and filtrates from CroV-infected BVI or E4-10 cultures, or with mavirus as a control, and monitored virophage DNA replication by qPCR. BVI-reactivated virophages replicated to levels comparable to mavirus, with an increase of more than five orders of magnitude at 3 dpi, whereas replication of E4-10-reactivated virophages was slightly less efficient than mavirus (Fig. 1*C*). No virophage propagation was observed in the absence of CroV.

In addition, negative staining transmission electron microscopy revealed the presence of virophage-like capsids in BVI filtrates (Fig. 1*E*). Although we found no virophage particles in E4-10 filtrates, such capsids were present after an additional round of propagation in CroV-infected RCC970 cultures (Fig. 1*F*), indicating low virion concentrations directly after reactivation in E4-10 cells. These results show that type 4 EMALEs from two different *Cafeteria* populations reactivate upon CroV infection and produce infectious virophage particles.

We then investigated whether other EMALE types also responded to CroV infection by sequencing total DNA from the 0.22-µm-filtered lysates. Unpackaged DNA was removed by DNase treatment prior to DNA extraction. BVI and E4-10 filtrates yielded sufficient amounts of DNA for Illumina sequencing, whereas RCC970 and Cflag filtrates contained insufficient DNA for sequencing. The sequence reads were aligned to their respective *C. burkhardae* genome assemblies (33), with 96% of reads from the BVI filtrate mapping against the BVI genome and 34% of reads from the E4-10 filtrate mapping against the E4-10 genome. When we determined the relative sequencing depth for each EMALE type, we found that almost all reads (99.4% for strain BVI and 99.9% for strain E4-10) mapped to type 4 EMALEs (Fig. 1*G* and *SI Appendix*, Table S1). Sequence matches to other EMALE types were random and involved fewer than 0.05% of reads.

Sequencing reads were then used to assemble genomes of reactivated virophages. Attempts to assemble full genomes of BVI virophages failed and resulted in short contigs, indicating a mixture of different type 4 EMALEs that caused assembly problems due to partial sequence overlap. In contrast, assembly of E4-10 reads produced a single contig with high sequence similarity to mavirus, and several contigs related to bacteria and bacteriophages that are likely derived from the mixed bacterial community present in *Cafeteria* sp. cultures, which serve as a food source for the phagotrophic flagellates (*SI Appendix*, Table S1). Detailed analysis of the mavirus-like sequence revealed two subpopulations that we named EV1 (E4-10 virophage 1) and EV2 (E4-10 virophage 2). These two virophage genomes were highly syntenic except for a gene encoding a FNIP/IP22 repeat protein (see Fig. 3*B*).

To distinguish the EV virophages in individual reaction experiments, we designed PCR primers for the FNIP/IP22 gene and conducted CroV infection experiments with E4-10 cells in five replicates. After cell lysis, the 0.22 μm filtrates were analyzed by PCR using EV1 and EV2 specific primers. Fig. 1*H* depicts how different biological replicates of CroV infections produced different amounts of EV1 and EV2, with some cultures reactivating only one but not the other virophage version (compare replicates 1, 2 and 4).

These experiments unambiguously show that CroV infection of *C. burkhardae* reactivates type 4 EMALEs and induces the production of infectious virophage particles. Interestingly, however, reactivation of endogenous virophages appears to be a stochastic and sometimes inefficient process.

### Reactivated Virophages Inhibit CroV Replication in Subsequent Rounds of Infection.

**Fig. 2.**
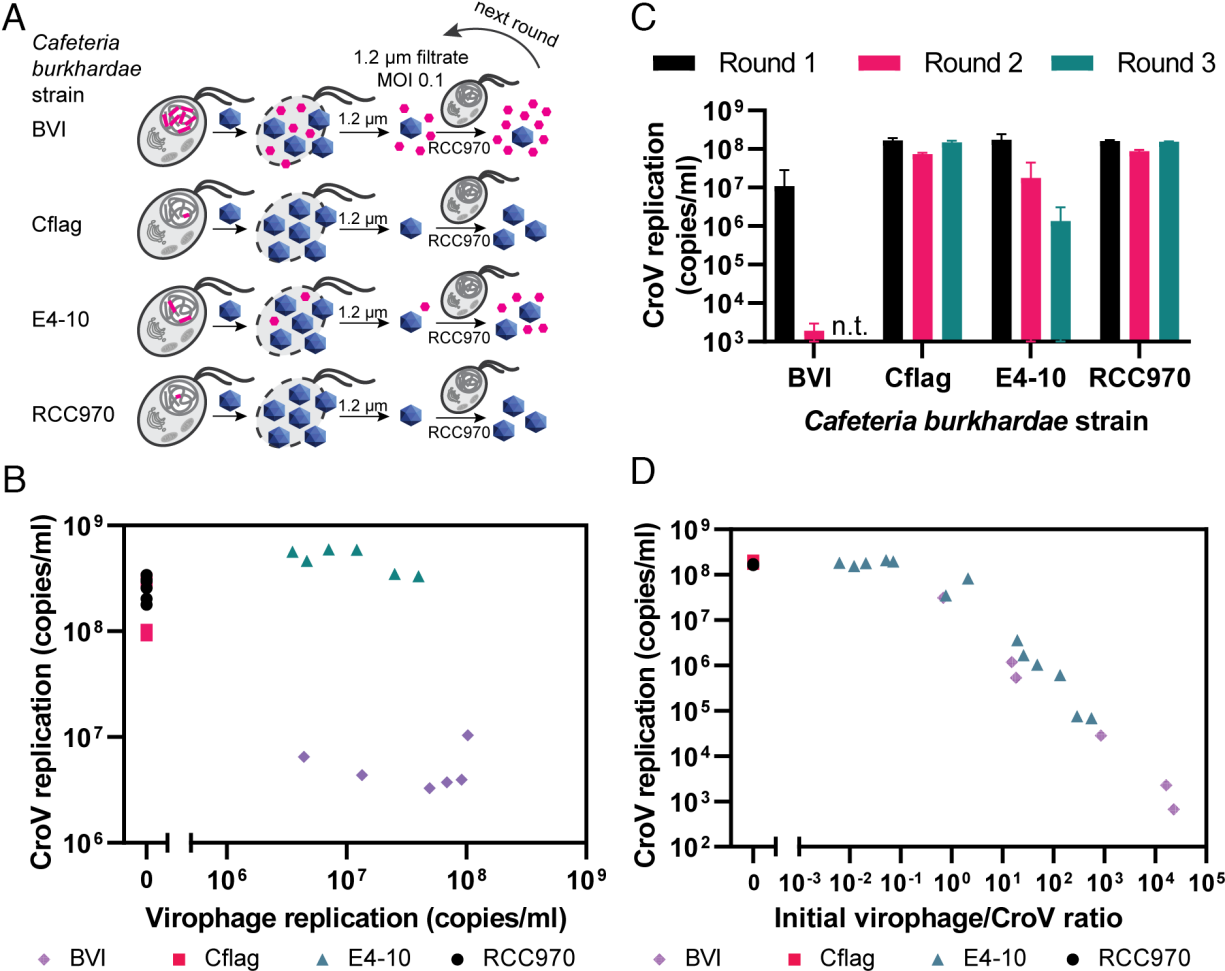
Reactivated virophages inhibit CroV replication in subsequent rounds of infection. (*A*) *C. burkhardae* strains BVI, Cflag, E4-10, and RCC970 were infected with CroV at MOI 0.1. After cell lysis, the cultures were 1.2 μm filtered to remove cell debris. CroV and virophages in the filtrates were analyzed by qPCR and used for three subsequent rounds of infection in *C. burkhardae* strain RCC970 to analyze the biological activity of reactivated virophages. After each round, the cultures were 1.2 µm filtered, and viral copy numbers were monitored. (*B*) Concentrations of CroV and virophage DNA in 1.2-μm filtrates after CroV infection of individual *C. burkhardae* strains. (*C*) CroV DNA replication levels during three consecutive rounds of infection in *C. burkhardae* strain RCC970 with 1.2-μm-filtered CroV that was produced in different *C. burkhardae* strains. (*D*) CroV DNA replication levels in relation to the virophage/CroV ratio at the start of infection. Host strains where the initial CroV infections took place are indicated; all subsequent infections were performed in host strain RCC970.

To test whether virophage reactivation interfered with CroV replication, we infected the four *C. burkhardae* strains with CroV and, following cell lysis, collected 1.2 μm filtrates containing both CroV particles and potential virophage particles (Fig. 2*A*). CroV and virophage genomes were quantified by qPCR to test for a potential link between the levels of virophage production and CroV replication. We observed that CroV replication was primarily dependent on the host strain (e.g., CroV replicating at low levels in strain BVI), and not on virophage production (Fig. 2*B*). This is in agreement with previous results showing that the induction of mavirus provirophages does not interfere with CroV replication (5). In the next step, we tested whether CroV, contained in lysates from different *C. burkhardae* strains that may also contain reactivated virophages, could replicate in strain RCC970, which lacks CroV-inducible EMALEs. We performed several rounds of infection with 1.2 μm-filtered CroV at MOI 0.1. For each infection round, we collected 1.2 μm filtrates at 3 dpi and monitored CroV and virophage replication using qPCR. Fig. 2*C* shows that replication of CroV produced from BVI and E4-10 strains was strongly inhibited in comparison with RCC970- and Cflag-produced CroV. CroV from BVI lysates showed decreased replication already during the first round of infection, whereas CroV produced on strain E4-10 replicated well during the first round of infection and decreased only in infection rounds two and three. Moreover, CroV from BVI lysates was essentially absent after the second round of infection, with levels barely above background that precluded a third round of infection.

We found a strong correlation between CroV replication levels and the ratio of virophage-to-CroV DNA at the beginning of each infection round. Fig. 2*D* shows that CroV replication was impaired when the approximate concentration of virophage genomes was equal to or higher than that of CroV genomes (ratio ≥ 1). These data indicate that virophages reactivated from wild-type *Cafeteria* populations inhibit CroV replication in a dose-dependent manner and can even stop the spread of giant virus infection entirely.

### Analysis of EMALE04 Clones Reveals High Strain-Level Diversity of Virophages.

Individual strains of *C. burkhardae* produced mixed virophage populations in response to CroV infection, which was particularly pronounced in strain BVI that harbors at least 16 EMALE04 loci in its genome. These virophage genomes share high similarity, except for a few unique genes that are present in certain virophages. To distinguish individual BVI virophages and to gain insight into the reactivation behavior of this host strain, we designed PCR primers specific for unique EMALE04 genes (*SI Appendix*, Fig. S3*A*). We then followed their DNA replication by qPCR analysis during four CroV infection experiments and found that each infection resulted in a different composition of virophages, reiterating the stochasticity of virophage reactivation (*SI Appendix*, Fig. S3*B*).

**Fig. 3.**
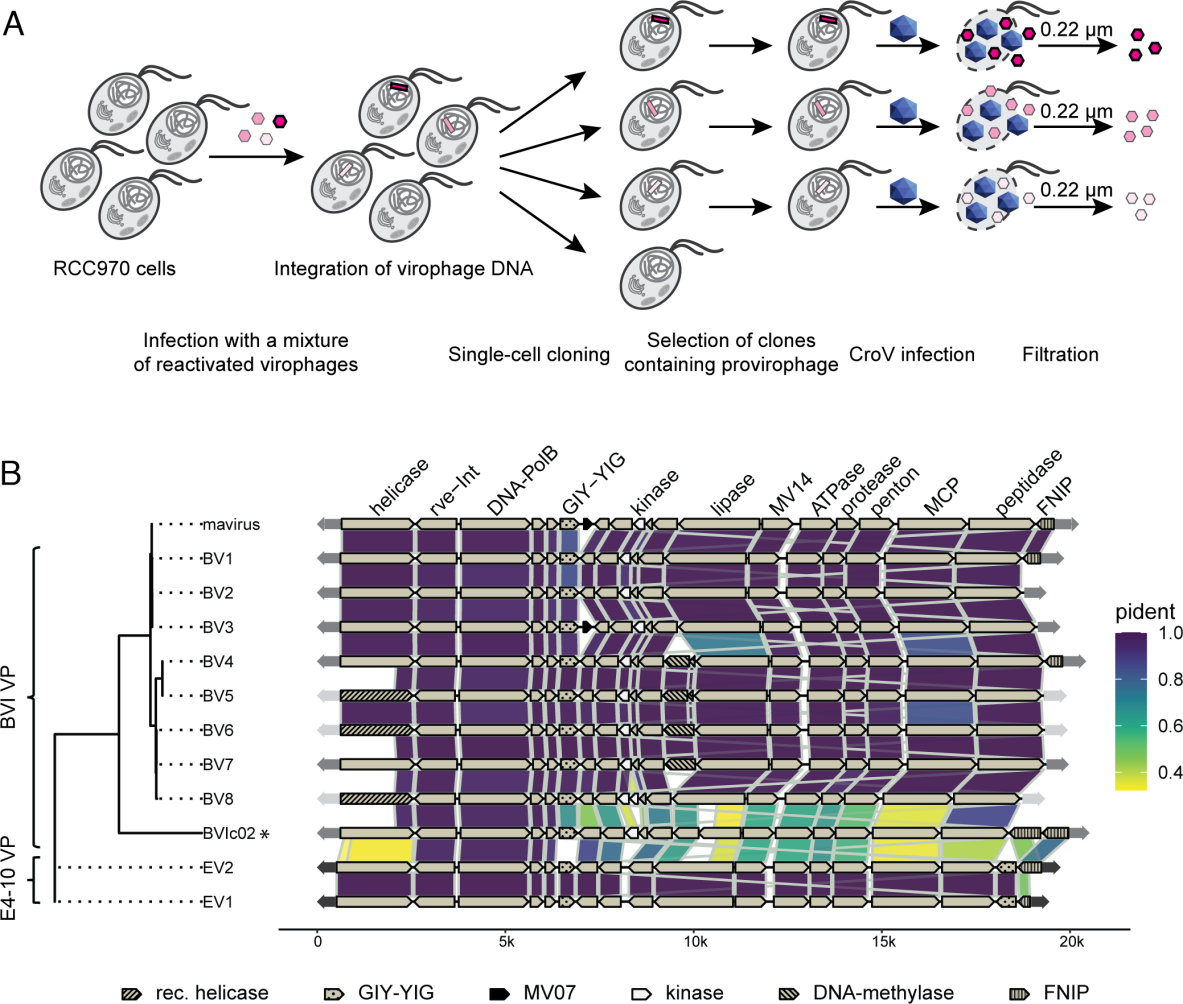
Cloning and comparative genomics of reactivated virophages from *C. burkhardae*. (*A*) Cloning strategy for reactivated virophages. *C. burkhardae* strain RCC970 was inoculated with a mixture of different virophages (shades of red), which led to virophage DNA integration into the cellular genome. Cells were cloned and clones were analyzed for the presence of provirophages. Positive clones were selected and infected with CroV to induce the production of integrated virophages. After cell lysis, cloned virophage populations were collected by filtration through a 0.22-μm filter. (*B*) Genome organization and comparison of clonal reactivated virophages. Each line represents a schematic genome diagram for one individual virophage. The reference mavirus genome is included for comparison. Virophage genomes are organized by a maximum likelihood tree which was constructed from multiple sequence alignments of the four virophage morphogenesis proteins: major capsid protein (MCP), penton protein, ATPase, and protease. * Virophage BVIc02 was not cloned; its sequence is derived from the BVI genome assembly.

To characterize individual virophages, we made use of their ability to integrate into nuclear host genomes. The workflow for obtaining clonal virophages is shown in Fig. 3*A* and started by inoculating the EMALE04-deficient RCC970 strain with a mixture of reactivated virophages, followed by a 1-wk-long incubation to allow the virophages to integrate. Copy numbers of integrated virophages genome were monitored by qPCR (using the ΔΔCt method, *SI Appendix*, Table S2), and cultures containing 0.1 to 0.3 provirophages per host genome were further processed, in order to minimize the probability of host cells carrying multiple integrations. We then performed limiting dilution cloning of the cells and selected clones that tested PCR-positive for virophages. Upon CroV infection and lysis, these cultures produced clonal populations of virophages. With this method, we were also able to cryopreserve virophages in their host-integrated state, which is not possible for free virophage particles as they lose infectivity after freeze-thawing.

Applying the cloning procedure above to lysates from the four CroV infections of host strain BVI (*SI Appendix*, Fig. S3*B*), we isolated eight different virophages named BV1 to BV8. The spectrum of virophage clones we obtained from the four infections was representative of the different types of virophages we had found in the mixed lysates (*SI Appendix*, Fig. S3*C*). To clone virophages from host strain E4-10, we started with infections where only EV1 or EV2 had reactivated (Fig. 1*H*) and selected one clone each for further characterization. The 10 clonal virophages were then propagated via CroV infection in host strain RCC970, virophage particles were concentrated by tangential flow filtration, and the resulting samples were analyzed by electron microscopy and DNA sequencing (see *Materials and Methods* for details).

Negative staining electron microscopy revealed virus-like particles in all clonal virophage preparations (*SI Appendix*, Fig. S4). The particles had hexagonal shapes with a diameter of 80.8 ± 5.5 nm and were indistinguishable from mavirus capsids. Given that all EMALEs encode a double-jelly roll major capsid protein, we conclude that virophage reactivation leads to the formation of nonenveloped capsids with icosahedral symmetry. These results are in agreement with previous reactivation studies on mavirus (5).

Genome sequencing of the EMALE04-derived virophage clones allowed us to analyze the strain-level diversity of this group of endogenous protist viruses (34). Overall, the 10 genomes were mostly syntenic to each other and to mavirus, yet each genome displayed unique features (Fig. 3*B*). Between the highly similar virophages EV1 and EV2 from *C. burkhardae* strain E4-10, only the FNIP/FGxxFN repeat-containing gene at the 3′ end of the genome differed significantly, whereas the rest of their genomes had only two single nucleotide polymorphisms. Interestingly, the FNIP/FGxxFN repeat, which belongs to the class of leucine-rich repeats, is prominently present in ≈70 CroV genes and the respective proteins are likely to mediate protein-protein interactions (35). The FNIP/FGxxFN repeats in mavirus, EV1/2, and some BVI virophages may have been acquired from CroV and could be implicated in the interaction of virophages with their giant host viruses, similar to what has been proposed for other types of host-related proteins such as collagen-like repeats in Sputnik and OLV (36). Although we did not observe phenotypic differences between EV1 and EV2, they may display slightly different characteristics when combined with other giant virus strains. Some EV1/2-encoded proteins such as Rve-Int, PolB, MV04, and MV05 shared more than 90% amino acid identity (AAI) with their mavirus homologs, whereas others such as Helicase, Lipase, and MCP had only 50 to 60% AAI compared to mavirus (Fig. 3*B*). Notably, the EV1/2 virophages contained two copies of the MV06-like GIY-YIG endonuclease.

Genome analysis of the eight BVI-derived virophages confirmed their PCR-based detection, although additional virophages may reactivate from this strain. For instance, DNA sequencing of 0.22-µm-filtered lysates yielded reads that mapped to a type 4 EMALE named BVIc02, which was fully assembled in the BVI genome, indicating reactivation of this element. However, BVIc02 production was too low for cloning (*SI Appendix*, Fig. S3*B*), possibly due to a frame-shift mutation in the predicted lipase gene causing a premature stop codon (Fig. 3*B*). Alternatively, BVIc02 may be ill-adapted to the CroV strain used here, as it is more distant to mavirus than the other BVI-derived virophages. BV1-BV8 displayed average nucleotide identities of 82 to 97% to mavirus, even though none of them shared the exact gene content with mavirus (Fig. 3*B*). In particular, we found three hypervariable regions. First, BVI virophages had three distinct versions of the predicted primase/helicase gene. The variability of this gene already became apparent during the initial genome analysis of *C. burkhardae* EMALEs and may indicate a higher recombination rate compared to other virophage genes (32). Interestingly, new primase/helicase versions were always associated with a change in the terminal inverted repeat sequence, suggesting a functional link between them. Second, we noticed that the central part of EMALE04 genomes is less conserved than its flanking regions that encode mostly core genes. The central region is typically composed of small ORFs, of which few have functional predictions such as DNA methylase and kinase domains. Apparently, these accessory genes are not essential for virophage replication but may provide benefits under certain conditions. The third hypervariable region is located at the 3′ terminus and involves the gene encoding FNIP/FGxxFN repeats (MV20 in mavirus). Overall, we observed five variants of FNIP/FGxxFN repeat-containing genes among reactivated virophages, in BV1/4 (identical to mavirus), EV1, EV2, and two such genes in BVIc02 (Fig. 3*B*).

### Reactivated Virophages Provide Host Protection against CroV Infection.

**Fig. 4.**
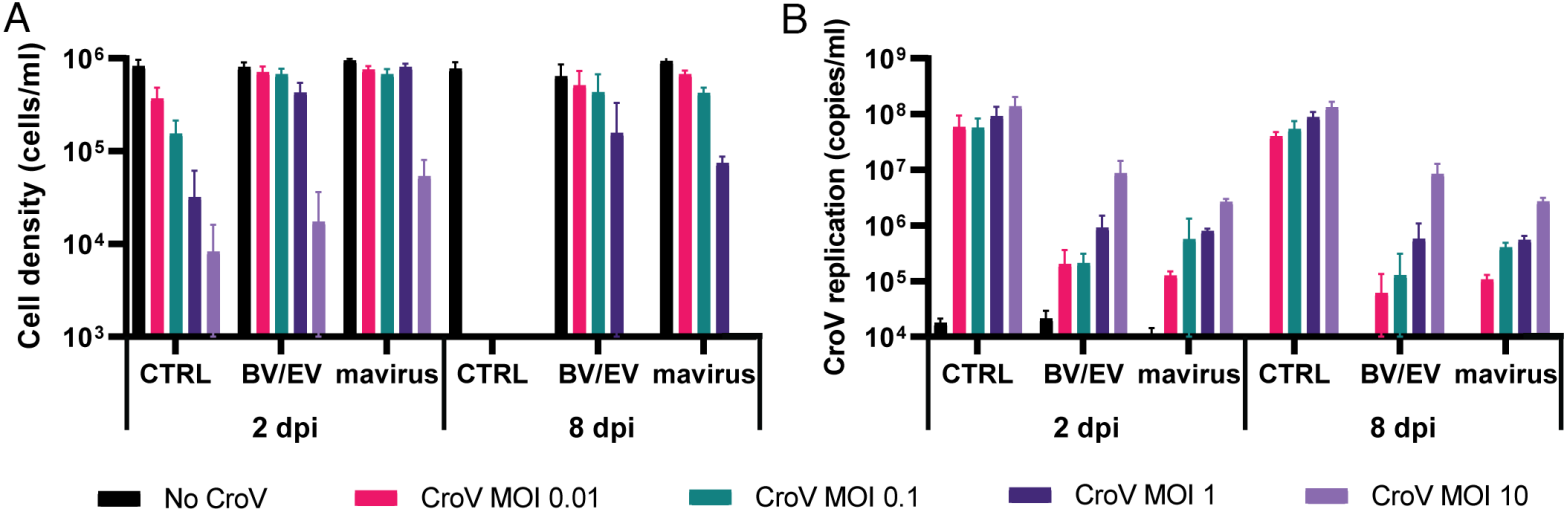
BV/EV virophages promote host cell survival by inhibition of CroV replication. *C. burkhardae* strain RCC970 was inoculated with individual clonal virophages at high MOI (20 to 50) and coinfected with CroV at different MOIs. Four different clonal virophages (BV4, BV7, BV8, and EV2) were individually tested, but here, we show their combined effect on cell host survival (*A*) and CroV replication (*B*) on days 2 and 8 postinfection. For detailed infection dynamics of individual virophages, see *SI Appendix*, Fig. S5. All experiments were performed in biological triplicates. Cell densities are based on microscopy counts; CroV concentrations were determined by qPCR. CTRL represents virophage-free CroV infections. Error bars represent SD.

Using the clonal strains of reactivated EMALE04 virophages, we compared their effects on CroV replication and host population survival. We performed CroV coinfection experiments with four selected virophages (BV4, BV7, BV8, and EV2) and with mavirus as a control. *C. burkhardae* strain RCC970 was inoculated with clonal virophages or mavirus at MOIs of 20 to 50 to ensure that every cell would be infected with at least one virophage particle. The same cultures were coinfected with CroV at MOIs of 0.01, 0.1, 1, or 10 and monitored daily for eight days by microscopy (host cells) and qPCR (CroV and virophages). The full infection dynamics for each virophage are provided in *SI Appendix*, Fig. S5. We found that all virophages inhibited CroV replication and increased host population survival when compared to virophage-free CroV infections (Fig. 4). Cell survival and CroV inhibition decreased with higher CroV MOIs, and at CroV MOI = 10 the cultures lysed irrespectively of whether virophages were present, indicating that virophages stop the spread of CroV by inhibiting the production of CroV virions rather than preventing lysis of CroV-infected cells. These results conform with previous reports of mavirus-CroV coinfection dynamics (5) and prove that virophages reactivated from naturally occurring endogenous elements are able to provide host population defense against a lytic giant virus.

### *C. burkhardae* Strains with Reactivable Endogenous Virophages (EMALE04) Are Geographically Widespread.

**Fig. 5.**
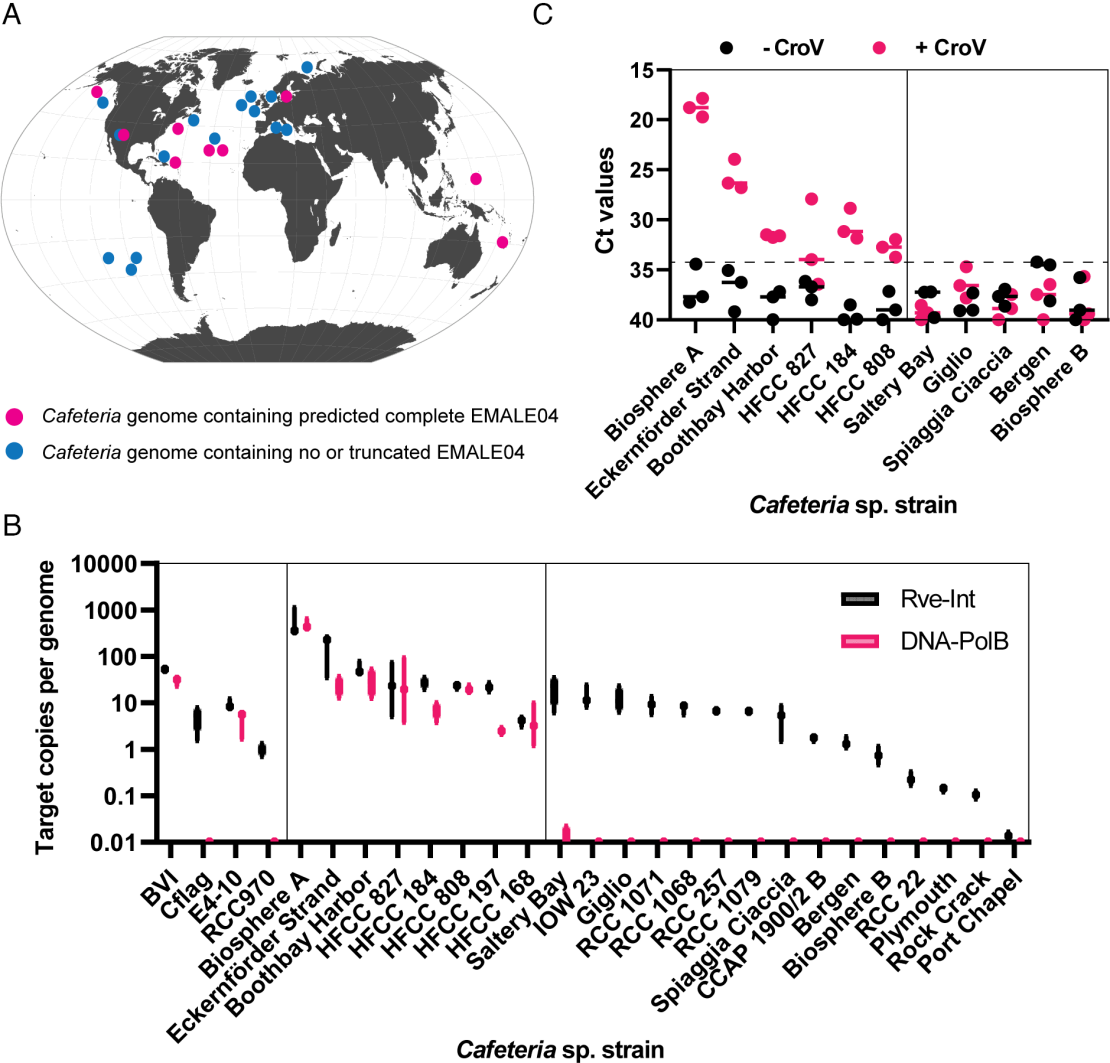
Global distribution of CroV-inducible endogenous virophages in heterotrophic flagellates of the genus *Cafeteria*. (*A*) Geographic origin of *Cafeteria* sp. strains used in this study. Prediction of genomes containing complete (pink dots) or truncated (blue dots) type 4 EMALEs is based on qPCR results. (*B*) Relative copy number of EMALE04 *rve-Int* and *polB* genes in different *Cafeteria* sp. genomes as determined by qPCR. (*C*) Quantification of type 4 EMALEs reactivation upon CroV infection (MOI = 0.1) of different *C. burkhardae* strains. The 0.22-μm-filtered lysates were treated with DNase prior to DNA release and qPCR analysis with primers specific to the *Rve-Int* gene of EMALE04. The dashed line indicates the background threshold.

Our analyses of only four *C. burkhardae* strains already revealed high variability in the genome content and reactivation potential of type 4 EMALEs. To further investigate the prevalence and activity of this group of virophages on a spatial scale, we tested 23 additional *Cafeteria* strains that had been isolated from various marine locations worldwide (Fig. 5*A* and *SI Appendix*, Table S3). We quantified type 4 EMALEs in these flagellate genomes with *rve-Int*- and *polB*-specific qPCR and found large differences among these two genes. While EMALE04-specific *rve-Int* genes were detected in almost all genomes, EMALE04-specific *polB* genes were found in only eight strains (Fig. 5*B*). We cannot exclude that our primer set may have failed to amplify some *polB* genes, even though the targeted region was highly conserved among all reactivated virophages. Therefore, we consider it more likely that the apparently higher prevalence of *rve-Int* may result from truncated or cryptic provirophages in the host genome, whereas the presence of both marker genes would be associated with complete provirophages. This hypothesis is in agreement with the observation that integrase genes are enriched in cryptic prophages (37). Furthermore, analysis of strains BVI, Cflag, E4-10, and RCC970 showed that in contrast to *rve-Int*, the *polB* gene was not amplified in strains Cflag and RCC970 (Fig. 5*B*). These two strains failed to reactivate virophages in response to CroV (Fig. 1*B*) and contained only truncated type 4 EMALEs, according to their respective genome assemblies (32).

We then selected six *polB*-positive *Cafeteria* strains with presumably intact type 4 EMALEs, and five *polB*-negative strains with presumably truncated type 4 EMALEs. We infected them with CroV, passed the culture supernatant through a 0.22-µm filter after cell lysis, and treated the filtrate with DNase prior to DNA extraction and *rve-Int*-specific qPCR analysis. EMALE04 DNA replication occurred only in those host strains that were predicted to contain intact EMALE04 genomes (Fig. 5*C*). In contrast, no evidence for EMALE04 reactivation was found in strains with presumably truncated type 4 EMALEs. Similar results were obtained when using *polB*-specific qPCR primers (*SI Appendix*, Fig. S6*A*). We noticed a positive correlation between the copy number of EMALE04 provirophages and their reactivation efficiency (compare Fig. 5*B* to Fig. 5*C* and *SI Appendix*, Fig. S6*A*).

We then investigated these six additional *Cafeteria* strains with functional type 4 EMALEs further by testing whether CroV replication is inhibited already during virophage reactivation, or during later coinfection cycles only. We infected the strains with CroV and measured the concentration of CroV- and virophage-specific DNA in the 1.2 μm-filtered cell lysates. Subsequently, we conducted several rounds of infection by inoculating host strain RCC970 with the six different 1.2 μm filtrates, as described in Fig. 2*A*. During virophage reactivation, we observed pronounced differences in CroV replication levels, ranging from 3E+07 copies per mL in strain Saltery Bay to 1.4E+09 copies/mL in strain HFCC827; however, there was no correlation between CroV replication and virophage reactivation (*SI Appendix*, Fig. S6*B*). In contrast, CroV was increasingly inhibited during subsequent rounds of coinfection when it originated from a virophage-producing host strain (*SI Appendix*, Fig. S6*C*). The level of inhibition depended on the virophage-to-CroV ratio at the start of the coinfection, with CroV replication levels dropping at ratios of 0.1 and higher (*SI Appendix*, Fig. S6*D*). These observations are in agreement with results from *C. burkhardae* strains BVI and E4-10 (Fig. 2) and previous reports of mavirus (5) and confirm that CroV replication is not impaired during virophage reactivation, but only during subsequent coinfection events with virophage particles. Serial propagation of CroV from the virophage nonproducer strains Saltery Bay and Spiaggia Ciaccia resulted in consistently high CroV DNA concentrations throughout four rounds of infection (*SI Appendix*, Fig. S6*C*).

In summary, our detailed characterization of host responses to CroV infection in 27 *C. burkhardae* strains revealed that type 4 EMALEs are not only geographically widespread but also represent functional endogenous virophages. We showed that CroV-induced virophage reactivation is common, but its efficiency varies between host strains and between individual infection experiments. All reactivated virophages were able to inhibit the spread of CroV infection upon further propagation, which highlights the ecological impact of virophages by controlling heterotrophic flagellate and giant virus populations in marine habitats.

### Reactivated Virophages Do Not Contain Inserted Ngaro Retrotransposons.

EMALEs in *C. burkhardae* are often interrupted by non-LTR retrotransposons of the Ngaro superfamily (32). In contrast, none of the reactivated virophage clones contained Ngaro sequences. DNA sequencing of reactivated virophages from strains E4-10 and BVI did not yield any reads mapping to full Ngaro elements either. The only exception were a few reads from BVI-reactivated virophages that aligned to Ngaro AB repeats which are typically located at the retrotransposon termini, implying that some virophages may contain remnants of Ngaro elements. Furthermore, we tried to match reactivated virophage sequences with specific Ngaro-interrupted EMALEs in *Cafeteria* genomes. Since most type 4 EMALEs were only partially assembled, we resequenced the genomes of host strains BVI and E4-10 with Nanopore technology to obtain long reads spanning the entire length of an EMALE (~20 kb) (38).

Long-read resequencing of the BVI genome allowed us to reconstruct the full genomes of three type 4 EMALEs that corresponded to the clonal virophages BV3, BV5, and BV7 (*SI Appendix*, Table S4). Another two elements matched to BV2 and BV8, although the endogenous and exogenous genomes were not identical. In these two cases, the EMALEs contained Ngaro elements that were not present after reactivation. In the E4-10 genome, we found fully assembled EMALEs matching the clonal virophages EV1 and EV2, with a retrotransposon in the EV2-matching EMALE. This observation indicates that Ngaros may be excised during the reactivation of EMALEs, and the functionality of Ngaro-containing EMALEs was already suggested after finding that their genes were not more fragmented than those of Ngaro-free EMALEs (32). To test whether we might have missed an Ngaro-free EMALE04 that could have led to the reactivation of virophage EV2 because it was not properly assembled, we examined the raw Nanopore reads of E4-10. We found no Ngaro-free reads that contained the EV2-specific FNIP repeats (*SI Appendix*, Fig. S7), supporting the hypothesis that EV2 reactivated from an endogenous element that contained a Ngaro retrotransposon and that was excised during reactivation.

## Discussion

We had previously shown that the virophage mavirus is able to integrate into host genomes and can be reactivated by CroV infection (5), and that naturally occurring endogenous virophages are abundant and diverse in *Cafeteria* populations (32). However, neither of these studies alone was sufficient to demonstrate the ecological importance of host-integrated virophages. In this work, we provide experimental evidence that endogenous virophages from wild protist populations are reactivated by giant virus infection and can produce infectious virions. Although the concentrations of reactivated virophages vary widely, subsequent coinfection events amplified the virophage response rapidly, leading to pronounced phenotypes in CroV inhibition and host cell survival. Our findings are in line with the virophage defense hypothesis, which states that endogenous virophages act as mutualists of their hosts by providing specific protection against lytic giant viruses (5, 30). While this statement cannot be generalized for other virus-protist systems, we now have strong evidence to support a virophage-based defense system against giant viruses in *C. burkhardae*.

Interestingly, only one of eight different EMALE types responded to CroV infection. EMALEs of type 4 are the closest known relatives to mavirus and, unlike other EMALE types, contain the conserved CroV late gene promoter motif. The transcriptional connection to CroV provides a plausible explanation why only EMALE04 virophages can be induced by CroV infection, whereas other EMALEs are probably specific for different types of giant viruses (32). Another possibility is that virophages interact with CroV through their shared FNIP/FGxxFN repeat domain-containing proteins.

The induction of a specific EMALE locus by an infecting giant virus is an inefficient process. Out of eight tested *Cafeteria* strains, five contained reactivable type 4 EMALEs that produced on average fewer than 10^5^ virophages per mL after CroV infection (based on qPCR). Such low virophage concentrations are difficult to detect by electron microscopy and therefore endogenous virophage reactivation can be easily overlooked. Replicate CroV infection experiments with the same host strain revealed that the reactivation of type 4 EMALEs is stochastic or may depend on the physiological state of the cell, resulting in varying mixtures of individual virophage strains. The chance of generating a virophage response is increased by the presence of multiple EMALE copies per host as shown by the observation that EMALE04 reactivation in host strain BVI with ≈16 copies was much more efficient than in host strain E4-10 with only two copies.

The biological activity of reactivated virophages from wild flagellate populations was comparable to that of the virophage mavirus by inhibiting CroV and increasing host population survival during subsequent rounds of infection in a dose-dependent manner (5). Therefore, endogenous virophages from wild *C. burkhardae* populations can provide efficient protection against CroV to their host populations. Another similarity to mavirus is that CroV replication was not inhibited during the initial round of infection when provirophage induction occurred, suggesting that this process is slower or less efficient than a coinfection with infectious mavirus particles. CroV can thereby still produce some progeny virions, which may ensure that mavirus-like virophages can retain their horizontal mode of transmission.

CroV-responsive endogenous virophages were present in wild *Cafeteria* populations from the Atlantic and Pacific Oceans and the Baltic Sea, proving that functional EMALEs are widespread in the marine environment. However, the reverse conclusion cannot be made, as the absence of EMALE04 virophage sequences in a particular flagellate strain does not imply their absence in an entire geographical region. First, the genetic composition of *Cafeteria* populations will change over time due to ocean currents and genetic drift. Second, our flagellate strains are clonal populations and thus reflect only a subset of the genetic diversity that is present at a particular location. As an example, we included two clonal *Cafeteria* strains that originated from the same water sample at the Biosphere 2 artificial ocean in Arizona, USA. Surprisingly, the EMALE04 content of these two clones varied dramatically, with strain Biosphere A having the highest copy number of complete type 4 EMALEs in this study (≈290) and high levels of reactivation, whereas strain Biosphere B contained probably only one cryptic EMALE04 that did not reactivate. The strain-level diversity and type composition of endogenous virophages in wild protist populations thus remains to be studied.

Even less is known about the geographical distribution of CroV and the strain-level diversity of *Cafeteria*-infecting viruses in the subfamily *Aliimimivirinae* (39). CroV was isolated in 1989 from coastal waters of the Gulf of Mexico (40), and since then, only one other study observed a large virus lysing *Cafeteria* during enrichment experiments in the Indian Ocean (41); however, no sequence information about this virus is available. Metagenomic studies indicate the presence of CroV-like sequences in various marine locations (42, 43), but isolation of additional giant viruses infecting *Cafeteria* is needed to study the basis of virophage - giant virus specificity.

Interestingly, our findings also have practical consequences for the isolation of novel giant viruses. If protists are able to mount a virophage defense in response to giant virus infection, the presence of a giant virus may go unnoticed in screening experiments that rely solely on host cell lysis or the appearance of cytopathic effects. Possible solutions to such virus isolation problems include flow cytometry detection of giant virus particles after short and long incubation periods, electrophoretic detection of free virophage genomes in the size range of 15 to 40 kbp, or testing multiple strains of a protist species of interest. Such considerations are surely not exaggerated, given that endogenous DNA viruses are much more common in unicellular eukaryotes than previously assumed. A recent study uncovered tens of thousands of virophages and PLVs in the genomes of all major eukaryotic supergroups (19). While their modes of action may be diverse and their relations to giant viruses remain unclear in most cases, many can be presumed to regulate the activity of giant viruses (22). The marine heterotrophic flagellate *Cafeteria burkhardae* is therefore exemplary for the still largely unexplored role of virophages in microbial ecology.

## Materials and Methods

### Host and Virus Strains.

The geographic location and isolation year of individual *Cafeteria* strains are depicted in *SI Appendix*, Table S3. After isolation, the cultures were single-cell cloned and continuously passaged approximately every 4 wk in f/2 enriched artificial seawater medium supplemented with one to three autoclaved wheat grains per 10 mL to stimulate bacterial growth. For infection experiments, cells were grown in f/2 enriched artificial seawater medium supplemented with 0.03% (w/v) Bacto yeast extract (Becton, Dickinson, Germany). Cultures were grown in flat-bottom 125 mL or 250 mL polycarbonate Erlenmeyer flasks (VWR, Germany) at room temperature. The viruses used for infection experiments were Cafeteria roenbergensis virus (CroV) strain BV-PW1 (44) and mavirus strain Spezl (4).

### Determination of Viral Titers.

The infectivity of CroV was measured by end point dilution assays described previously (5). The statistical method by Reed and Muench (45) was used to determine the 50% end point. When we performed several rounds of infection of CroV in order to analyze the effect of reactivated virophages on CroV replication, we determined only the approximate titer of CroV by qPCR detection of CroV gDNA. For mavirus and isolated virophages, end point dilution assays could not be employed because a productive virophage infection does not cause cell lysis. Therefore, we used it for the detection of approximate virophage titer qPCR measurement of virophage gDNA. However, based on our experience, the number of infectious particles can be 5 to 10 times lower than the amount of viral gDNA.

### Infection Experiments.

Infection experiments were performed in a similar way as described previously (5). *Cafeteria* cultures were propagated until they reached a density of >10^6^ cells per mL and before the infection the cultures were diluted with f/2 medium containing 0.03% (w/v) yeast extract to a cell density of 6 to 8 × 10^5^ cells per mL. Depending on the experiment, aliquots of 25 mL or 50 mL were inoculated with virus stock. The amount of the CroV/virophage inoculum varied between different infection experiments according to the desired MOI and the titer of the CroV/virophage working stock. CroV inoculum was stored at 4 °C and replaced every few months. Mavirus/virophage inoculum was always prepared fresh by CroV infection of a *Cafeteria* clone containing an integrated form of virophage, stored at 4 °C, and used within 2 wk.

When we analyzed the effect of reactivated virophages on CroV replication. We used 1.2-μm filtrates gained after CroV infection of individual *C. burkhardae* strains (*Collection of Reactivated Virophages*), which should contain both CroV and potentially reactivated virophages. We determined the amount of CroV gDNA by qPCR and performed several rounds of infection of *Cafeteria* strain RCC970. The *Cafeteria* culture was diluted to a density of 7 × 10^5^ cells per mL, 2 mL were distributed to each well of 12-well plate and infected with individual 1.2 μm filtered CroV suspension at an MOI of 0.1. After 2 d, each sample was filtered through a 1.2-μm syringe filter, analyzed by qPCR, and used for the next round of infection. For each *C. burkhardae* strain, we performed the experiment in biological triplicates.

Cell concentrations were measured by staining a 10 μL aliquot of the suspension culture with 1 μL of Lugol’s acid iodine solution and counting the cells on a hemocytometer (Neubauer Improved Counting Chamber, VWR Germany). Aliquots of 25 μL for qPCR analysis were sampled at appropriate time points and were immediately frozen and stored at −20 °C until further processing. All infections were performed in triplicates or quadruplicates.

### Collection of Reactivated Virophages.

Different *Cafeteria* strains were infected with CroV, and on the day when lysis was detected (at least 90% of cells died), the culture was passed through a syringe filter of 0.22-μm pore size (Techno Plastic Products), for collection of virophages, or filter of 1.2-μm pore size (Whatman) for collection of CroV together with virophages. The day of lysis varied between different strains and was dependent on CroV MOI. On the same day, filtrate from uninfected culture was collected as a control. To completely remove CroV, it was necessary to filter the lysate twice through 0.22-μm-pore-size filter. Virus stocks were stored at 4 °C; 25 μL aliquots were sampled for qPCR analysis and processed immediately.

### Sample Preparation for qPCR Analysis.

Samples containing cells and viruses were lysed before qPCR analysis to release DNA. Twenty-five microliters of suspension culture was mixed with 25 μL of water and 50 μL of 2× lysis buffer (20 mM Tris-HCl, pH 8.0; 2 mM EDTA; 0.002% Triton X-100; 0.002% SDS; 2 mg/mL proteinase K) in a PCR tube. The tubes were incubated at 58 °C for 1 h, and then, the protease was heat-inactivated at 95 °C for 10 min. Filtrates were treated with DNase before DNA extraction to remove unpackaged DNA. Twenty-five microliters of filtrate was mixed with 24 μL of water and 1 μL of TURBO DNase (Thermo Fisher Scientific) in a PCR tube. The tube was incubated at 37 °C for 30 min. Consequently, the sample was mixed with a 2× lysis buffer and treated the same way as suspension culture samples. Samples were stored at −20 °C. In order to analyze integrated virophages/EMALEs in the *Cafeteria* genome, we either extracted the DNA from cells or lysed only the cellular fraction to increase the amount of DNA. 200 to 500 µL of the cell culture was transferred to a 1 mL Eppendorf tube and centrifuged at 5,000 *g* for 5 min or 4,000 *g* for 10 min. Clones in 96-well plates were centrifuged directly in plates. The supernatant was quickly removed via aspiration. Afterward, the cell pellets were washed with PBS, resuspended in 100 µL of 1× lysis buffer (10 mM Tris-HCl, pH 8.0; 1 mM EDTA; 0.001% Triton X-100; 0.001% SDS; 1 mg/mL proteinase K, 0.2 mM CaCl_2_), and transferred to PCR tubes. The tubes were incubated at 58 °C for 1 h and heat-inactivated at 95 °C for 10 min.

### qPCR Analysis.

DNA target sequences were quantified by qPCR using the SYBR-related EvaGreen dye. Two microliters of analyzed samples was used as a template in a 20-μL qPCR reaction containing 10 µL of 2× Fast-Plus EvaGreen Master Mix with low ROX dye (Biotium, Inc. via VWR, Germany), 10 pmol of each forward and reverse primer (*SI Appendix*, Table S5), and 7.8 µL of ddH_2_O. No-template controls (NTC) contained ddH_2_O instead of an analyzed sample. Each qPCR reaction was performed in technical duplicates or triplicates. The Ct values of the NTC controls were consistently above 35, and therefore, Ct values above 35 were considered as a background. Thermal cycling was performed in a Stratagene Mx3005P qPCR system (Agilent Technologies, Germany) with the following settings: 95 °C for 5 min, 40 cycles of 95 °C for 10 s followed by 61 °C for 25 s and 72 °C for 25 s, a single cycle of 72 °C for 5 min, and a final dissociation curve was recorded from 50 °C to 95 °C. Mavirus gDNA was detected by primers Spezl-qPCR-5 and Spezl-qPCR-6 (*SI Appendix*, Table S5), which target the *MV18* MCP gene, and CroV gDNA was detected by primers CroV-qPCR-9 and CroV-qPCR-10 which target *crov283* gene. Reactivated virophages (including mavirus) were detected by Mav-Pol-fw and Mav-Pol-2-rv primers targeting conserved region of *DNA-PolB* gene or Int-2-fw and Int-2-rv primers targeting conserved region of *rve-INT* gene. Primers specific for selected virophage genes which were used to distinguish individual reactivated virophages are depicted in *SI Appendix*, Table S5 and the position of these primers is indicated in *SI Appendix*, Fig. S3. Primers were designed based on partial EMALE04 sequences found in BVI genome assembly. Selected type 4 EMALEs were aligned and variable sequences (later annotated as specific genes) were used as targets for qPCR primer design. Viral gDNA copies of mavirus and CroV were absolutely quantified using a standard curve, which was prepared by a tenfold dilution series that ranged from 10^1^ to 10^8^ molecules of a linearized pEX-A plasmid (Eurofins Genomics, Germany) carrying inserted respective PCR products, as described previously (5). Standard curve for absolute quantification of reactivated virophages was prepared in a similar way. PCR product amplified using primers Mav-Pol-fw and Mav-Pol-2-rv primers was inserted in a pCR4Blunt-TOPO vector (Thermo Fisher Scientific), linearized, and diluted to form a series from 10^1^ to 10^8^ molecules. The number of virophage gene copies per *Cafeteria* genome was determined by relative quantification. Target virophage sequence was amplified using Mav-Pol-fw and Mav-Pol-2-rv primers or Int-2-fw and Int-2-rv primers. As a reference, *Cafeteria* gene aspartyl-tRNA synthetase (*AspRS*) was amplified using Cr_E4-10-AspRS-fw and -rv primers. The number of virophage gene copies per *Cafeteria* genome was calculated by delta delta Ct method. The results were related to a sample containing gDNA from *Cafeteria* clone RCC970 8-8, which harbors one mavirus copy per genome.

### Cloning of Individual Reactivated Virophages.

Individual virophages were cloned via host cell genome integration and single-cell cloning. The cloning strategy is depicted in Fig. 3*A*. RCC970 strain of *C. burkhardae*, which does not contain any CroV-reactivable EMALEs, was inoculated with a mixture of reactivated virophages. Mixtures of reactivated virophages gained after individual CroV infections of BVI strain had sufficient titer and they were used directly for inoculation of RCC970 strain. To clone EV-derived virophages, we collected the virophages from infections, when only EV1 or EV2 reactivated, propagated EV1/EV2 in RCC970 strain coinfected with CroV to get sufficient virophage titer and inoculated RCC970 strain without CroV to integrate virophage DNA copies in the genome. After inoculation, the culture was propagated for 1 wk to provide time for virophage integration. Every second day 1 mL of culture was transferred to a new flask containing 20 mL of fresh medium. After 1 wk, the amount of integrated virophage copies per genome was monitored using qPCR. Five hundred microliters of cell suspension was lysed and analyzed by qPCR (ΔΔ Ct method). Cultures containing from 0.1 to 0.3 provirophages per genome were consequently processed. These cultures should contain integrated virophage in every third to tenth cell, and therefore, the probability of integration of several virophage copies in one genome should be low. Cell clones were isolated using single-cell dilution. The cell cultures were diluted with f/2 medium + 0.03% (w/v) yeast extract to a concentration of 1.5 cells/mL (0.3 cells/200 µL). Each well of a 96-well plate was filled with 200 µL of the diluted cell culture so that, on average, every third well received one cell. After 1 wk in room temperature, the wells that contained single cell cultures were selected and transferred to a new 96-well culture plate. The clonal cultures were propagated every week by taking 10 µL of each culture and transferring it to a new well plate containing 200 µL fresh medium per well. Clones were screened by qPCR. Two hundred microliters of clonal cultures was lysed prior qPCR analysis (*Sample Preparation for qPCR Analysis*). Clones containing integrated virophage gDNA were selected based on positive signal (Ct value lower than 35) using Mav-Pol-fw and Mav-Pol-2-rv primers. Consequently, positive clones were analyzed by qPCR using primers targeting specific virophage genes to distinguish individual virophages. For each individual virophage, we picked one or two cell clones, which was used for clonal virophage production and storage. *C. burkhardae* cultures can be frozen in contrast to virophage particles, which completely lose infectivity after freezing.

### Production of Virophages from Clonal Cell Cultures.

*Cafeteria* cell clones containing integrated virophages were infected with CroV (MOI 0.1 or 0.01), and on the day when lysis was detected (at least 90% of cells died), the culture was passed through a syringe filter of 0.22-µm pore size. Filtrates were lysed and analyzed by qPCR using Mav-Pol-fw and Mav-Pol-2-rv primers to determine the amount of reactivated virophages. Usually, the production of virophages after reactivation was low, and therefore, one round of virophage propagation was necessary. *Cafeteria* RCC970 strain was coinfected with virophage at MOI 0.01 or lower and CroV MOI 0.1. After cell lysis, 0.22-μm filtrate was collected and analyzed by qPCR.

### Concentration of Virophage Particles for Electron Microscopy and DNA Sequencing.

Samples of reactivated (mixed) virophages were prepared by CroV infection of individual *C. burkhardae* (BVI, C-flag, E4-10, RCC970) strains. Four liters of *Cafeteria* cultures was infected with CroV at MOI 0.1 to 0.01. After cell lysis, the cultures were centrifuged at 7,000 *g* for 40 min at 4 °C and filtered through 0.22-μm-pore-size filter unit (PES, Millipore Stericup). The filtrates were then concentrated on ice with a 100,000 MWCO PES Vivaflow 200 tangential flow filtration unit (Sartorius via VWR, Germany) to a final volume of approximately 20 mL. The concentrates were passed through a 0.1-μm pore-size PVDF Millex syringe filter (Millipore). Twenty microliters of these samples was analyzed by electron microscopy. The rest was ultracentrifuged at 28,000 rpm for 3 h at 4 °C using a SW28 rotor (Beckman Coulter, Germany) in a Beckman Optima ultracentrifuge. The pellet was resuspended in 175 μL PBS, mixed with 20 μL of 10× TURBO DNase buffer, and treated with 5 μL of TURBO DNase at 37 °C for 30 min. DNA was extracted by the QIAamp DNA MINI kit (Qiagen) following the manufacturer’s instructions. Samples of cloned virophages were prepared by coinfection of RCC970 strain. Two liters of *Cafeteria* strain RCC970 was coinfected with cloned virophage at MOI 0.01 to 0.001 and CroV at MOI 0.1. Consequently, the samples were processed the same way as described above for samples containing mixtures of reactivated virophages.

### Cellular gDNA Extraction.

In order to isolate gDNA for qPCR 10 to 50 mL of individual Cafeteria spp. cultures we centrifuged at 4,500 *g* for 10 min, pellets were washed with PBS and resuspended in 200 μL PBS. DNA was extracted by the QIAamp DNA MINI kit (Qiagen) following the manufacturer’s instructions and diluted to concentration 50 to 100 ng/μL.

### Virophage DNA Sequencing, Assembly, and Annotation.

Genomes of reactivated virophages from E4-10 and BVI strains were sequenced at the Max Planck Genome Centre (Cologne, Germany). Two million reads (2 × 250 bp) were produced on the Illumina HiSeq2500 platform. The reads were mapped with minimap2 v2.22 (46) to previously published assemblies of the respective host with annotated integrated virophages (33). Coverage of integrated virophages was determined with “samtools bedcov” v1.9 (47) and aggregated with a custom R script.

Cloned virophages BV1, BV3, BV4, BV5, BV6, and BV7 were sequenced at Starseq, Germany (NextSeq2000, 5 mil. reads per sample), and virophages EV1, EV2, BV2, and BV8 at Eurofins, Germany (INVIEW virus sequencing, 5 mil. reads per sample). Virophage genomes were assembled using Geneious software and manual finishing. Coding DNA sequences were predicted using GeneMarkS (48), and functional annotation was carried out using BLASTp (49) searches against the nonredundant protein collection of the NCBI with manual curation to produce high-quality annotation files.

### Virophage Synteny and Phylogeny Analysis.

To compare the genome organization of the reactivated virophages genomes, we computed all-versus-all protein alignments with MMseqs2 v13-45111 (50) and visualized them with gggenomes (https://github.com/thackl/gggenomes). Phylogenetic relationships were determined based on a concatenated multiple sequence alignments of the morphogenesis proteins (MCP, penton, ATPase, and protease) computed with Mafft v7.490 (51). A joint phylogenetic tree was reconstructed with FastTree v2.1.11a (52).

### Host Nanopore Resequencing and Assembly.

gDNA from *Cafeteria burkhardae strain* E4-10 was isolated and sequenced at Max Planck Genome Centre (Cologne, Germany) using a GridION X5. High-molecular-weight gDNA from BVI strain was isolated using the Blood & Cell Culture DNA Mini Kit (Qiagen, Hilden, Germany). The SQK-LSR109 kit was used to prepare a DNA library, which was sequenced on a MinION R9.4.1 SpotON Flow Cell (Oxford Nanopore Technologies, UK). A draft assembly was generated with Flye v2.9.1 (53) from reads longer than 4 kb with default settings.

### Electron Microscopy.

Aliquots (≈3 μL) of the concentrated samples were incubated for 2 min on Formvar/Carbon coated 75 mesh Cu grids (Plano, Germany) that had been hydrophilized by glow discharge. Grids were rinsed with ddH_2_O, stained for 90 s with 1% uranyl acetate, and imaged on a Tecnai T20 electron microscope (FEI, USA) with an acceleration voltage of 200 kV.

### PCR of EV1/EV2 FNIP/FG Repeats.

A culture of *Cafeteria burkhardae* strain E4-10 was diluted to a density 7 × 10^5^ cells per mL, and 25 mL aliquots were distributed to five individual flasks. Flasks 1-3 were infected with CroV at M0I 0.1 and flasks 4 and 5 with CroV at MOI 0.01. Three days postinfection, when the majority of cells had lysed, the cultures were filtered through a syringe filter of 0.22-um pore size. Twenty-five microliters of each filtrate was lysed (*Sample Preparation for qPCR Analysis*), and 2 μL of lysate was used as a template in a PCR reaction to distinguish EV1 and EV2. The region containing FNIP/FG repeats was amplified in 25 μL reaction mix containing 2 μL template, 2.5 μL Ex Taq Buffer, 0.6 U of TaKaRa Ex Taq Hot Start Polymerase (Takara), 0.2 mM dNTPs, and 0.4 μM of primers FG-fw and FG-rv (*SI Appendix*, Table. S5). The PCR was performed in a thermocycler with the following cycling conditions: 1 min denaturation at 98 °C; 35 cycles of 10 s denaturation at 98 °C, 30 s annealing at 60 °C and 1 min extension at 72 °C; and a final 5 min extension at 72 °C. For product analysis, 5 μL of each reaction was mixed with loading dye and pipetted on a 1% (w/v) agarose gel supplemented with GelRed. The marker lanes contained 0.5 μg of GeneRuler 100 bp DNA Ladder (Fermentas, Thermo Fisher Scientific, USA). The gel was electrophoresed at 90 V for 1 h and visualized using a ChemiDoc MP Imaging system (Bio-Rad).

## Supplementary Material

Appendix 01 (PDF)

## Data Availability

Raw sequencing reads obtained from mixed and cloned reactivated viral particles, Nanopore-resequenced hosts, and assemblies generated from reactivated clones have been deposited at the European Nucleotide Archive under project accession PRJEB61642 (38). Exogenous EMALE04 virophage sequences BV1-8 and EV1-2 are also available from https://zenodo.org/records/10377179 (34).

## References

[1] S. Roux , Updated virophage taxonomy and distinction from polinton-like viruses. Biomolecules **13**, 204 (2023). 36830574 10.3390/biom13020204 PMC9952930

[2] S. Duponchel, M. G. Fischer, Viva lavidaviruses! Five features of virophages that parasitize giant DNA viruses. PLoS Pathog. **15**, e1007592 (2019). 30897185 10.1371/journal.ppat.1007592 PMC6428243

[3] B. La Scola , The virophage as a unique parasite of the giant mimivirus. Nature **455**, 100–104 (2008). 18690211 10.1038/nature07218

[4] M. G. Fischer, C. A. Suttle, A virophage at the origin of large DNA transposons. Science **332**, 231–234 (2011). 21385722 10.1126/science.1199412

[5] M. G. Fischer, T. Hackl, Host genome integration and giant virus-induced reactivation of the virophage mavirus. Nature **540**, 288–291 (2016). 27929021 10.1038/nature20593

[6] S. Mougari , A virophage cross-species infection through mutant selection represses giant virus propagation, promoting host cell survival. Commun. Biol. **3**, 248 (2020). 32439847 10.1038/s42003-020-0970-9 PMC7242381

[7] J. Zhou , Diversity of virophages in metagenomic data sets. J. Virol. **87**, 4225–4236 (2013). 23408616 10.1128/JVI.03398-12 PMC3624350

[8] S. Roux , Ecogenomics of virophages and their giant virus hosts assessed through time series metagenomics. Nat. Commun. **8**, 858 (2017). 29021524 10.1038/s41467-017-01086-2 PMC5636890

[9] D. Paez-Espino , Diversity, evolution, and classification of virophages uncovered through global metagenomics. Microbiome **7**, 1–14 (2019). 31823797 10.1186/s40168-019-0768-5 PMC6905037

[10] S. Yau , Virophage control of antarctic algal host-virus dynamics. Proc. Natl. Acad. Sci. U.S.A. **108**, 6163–6168 (2011). 21444812 10.1073/pnas.1018221108 PMC3076838

[11] D. Wodarz, Evolutionary dynamics of giant viruses and their virophages. Ecol. Evol. **3**, 2103–2115 (2013). 23919155 10.1002/ece3.600 PMC3728950

[12] B. P. Taylor, M. H. Cortez, J. S. Weitz, The virus of my virus is my friend: Ecological effects of virophage with alternative modes of coinfection. J. Theor. Biol. **354**, 124–136 (2014). 24662503 10.1016/j.jtbi.2014.03.008

[13] E. V. Koonin, M. Krupovic, Polintons, virophages and transpovirons: A tangled web linking viruses, transposons and immunity. Curr. Opin. Virol. **25**, 7–15 (2017). 28672161 10.1016/j.coviro.2017.06.008 PMC5610638

[14] J. G. N. Barreat, A. Katzourakis, Phylogenomics of the Maverick virus-like mobile genetic elements of vertebrates. Mol. Biol. Evol. **8**, 1731–1743 (2021). 10.1093/molbev/msaa291 PMC8097293 33481003

[15] V. V. Kapitonov, J. Jurka, Self-synthesizing DNA transposons in eukaryotes. Proc. Natl. Acad. Sci. U.S.A. **103**, 4540–4545 (2006). 16537396 10.1073/pnas.0600833103 PMC1450207

[16] E. J. Pritham, T. Putliwala, C. Feschotte, Mavericks, a novel class of giant transposable elements widespread in eukaryotes and related to DNA viruses. Gene **390**, 3–17 (2007). 17034960 10.1016/j.gene.2006.08.008

[17] M. Krupovic, D. H. Bamford, E. V. Koonin, Conservation of major and minor jelly-roll capsid proteins in Polinton (Maverick) transposons suggests that they are bona fide viruses. Biol. Direct **9**, 6 (2014). 24773695 10.1186/1745-6150-9-6 PMC4028283

[18] N. Yutin, S. Shevchenko, V. Kapitonov, M. Krupovic, E. V. Koonin, A novel group of diverse Polinton-like viruses discovered by metagenome analysis. BMC Biol. **13**, 95 (2015). 26560305 10.1186/s12915-015-0207-4 PMC4642659

[19] C. Bellas , Large-scale invasion of unicellular eukaryotic genomes by integrating DNA viruses. Proc. Natl. Acad. Sci. U.S.A. **120**, e2300465120 (2023). 37036967 10.1073/pnas.2300465120 PMC10120064

[20] C. M. Bellas, R. Sommaruga, Polinton-like viruses are abundant in aquatic ecosystems. Microbiome **9**, 13 (2021). 33436089 10.1186/s40168-020-00956-0 PMC7805220

[21] G. Blanc, L. Gallot-Lavallée, F. Maumus, Provirophages in the Bigelowiella genome bear testimony to past encounters with giant viruses. Proc. Natl. Acad. Sci. U.S.A. **112**, E5318–E5326 (2015). 26305943 10.1073/pnas.1506469112 PMC4586850

[22] S. Roitman , Isolation and infection cycle of a polinton-like virus virophage in an abundant marine alga. Nat. Microbiol. **8**, 332–346 (2023). 36702941 10.1038/s41564-022-01305-7

[23] E. E. Chase, C. Desnues, G. Blanc, Integrated viral elements suggest the dual lifestyle of Tetraselmis spp. Polinton-like viruses. Virus Evol. **8**, veac068 (2022). 35949392 10.1093/ve/veac068 PMC9356565

[24] S. Santini , Genome of Phaeocystis globosa virus PgV-16T highlights the common ancestry of the largest known DNA viruses infecting eukaryotes. Proc. Natl. Acad. Sci. U.S.A. **110**, 10800–10805 (2013). 23754393 10.1073/pnas.1303251110 PMC3696832

[25] A. Pagarete, T. Grébert, O. Stepanova, R. Sandaa, G. Bratbak, Tsv-N1: A novel DNA algal virus that infects Tetraselmis striata. Viruses **7**, 3937–3953 (2015). 26193304 10.3390/v7072806 PMC4517135

[26] M. Gaia , Zamilon, a novel virophage with mimiviridae host specificity. PLoS One **9**, e94923 (2014). 24747414 10.1371/journal.pone.0094923 PMC3991649

[27] Y. Sheng, Z. Wu, S. Xu, Y. Wang, Isolation and identification of a large green alga virus (Chlorella Virus XW01) of Mimiviridae and its virophage (Chlorella Virus Virophage SW01) by using unicellular green algal cultures. J. Virol. **96**, e0211421 (2022). 35262372 10.1128/jvi.02114-21 PMC9006914

[28] M. Legendre , mRNA deep sequencing reveals 75 new genes and a complex transcriptional landscape in Mimivirus. Genome Res. **20**, 664–674 (2010). 20360389 10.1101/gr.102582.109 PMC2860168

[29] S. Mougari , Guarani virophage, a new Sputnik-like isolate from a Brazilian lake. Front. Microbiol. **10**, 1003 (2019). 31130943 10.3389/fmicb.2019.01003 PMC6510173

[30] M. Berjón-Otero, A. Koslová, M. G. Fischer, The dual lifestyle of genome-integrating virophages in protists. Ann. N. Y. Acad. Sci. **1447**, 97–109 (2019). 31162694 10.1111/nyas.14118

[31] E. V. Koonin, M. Krupovic, Virology: A parasite’s parasite saves host’s neighbours. Nature **540**, 204–205 (2016). 27929010 10.1038/540204a

[32] T. Hackl, S. Duponchel, K. Barenhoff, A. Weinmann, M. G. Fischer, Virophages and retrotransposons colonize the genomes of a heterotrophic flagellate. Elife **10**, e72674 (2021). 34698016 10.7554/eLife.72674 PMC8547959

[33] T. Hackl , Four high-quality draft genome assemblies of the marine heterotrophic nanoflagellate Cafeteria roenbergensis. Sci. Data **7**, 29 (2020). 31964893 10.1038/s41597-020-0363-4 PMC6972860

[34] T. Hackl, thackl/reactivated-endogenous-virophages: reactivated-endogenous-virophages-v1.1. Zenodo. https://zenodo.org/records/10377179. Deposited 14 December 2023.

[35] T. Huyton, M. Jaiswal, W. Taxer, M. Fischer, D. Görlich, Crystal structures of FNIP/FGxxFN motif-containing leucine-rich repeat proteins. Sci. Rep. **12**, 16430 (2022). 36180492 10.1038/s41598-022-20758-8 PMC9525666

[36] N. Yutin, D. Raoult, E. V. Koonin, Virophages, polintons, and transpovirons: A complex evolutionary network of diverse selfish genetic elements with different reproduction strategies. Virol. J. **10**, 158 (2013). 23701946 10.1186/1743-422X-10-158 PMC3671162

[37] A. Khan, A. R. Burmeister, L. M. Wahl, Evolution along the parasitism-mutualism continuum determines the genetic repertoire of prophages. PLoS Comput. Biol. **16**, e1008482 (2020). 33275597 10.1371/journal.pcbi.1008482 PMC7744054

[38] T. Hackl, reactivated endogenous virophages. European Nucleotide Archive. www.ebi.ac.uk/ena/browser/view/PRJEB61642. Deposited 24 April 2023.

[39] F. O. Aylward , Taxonomic update for giant viruses in the order Imitervirales (phylum Nucleocytoviricota). Arch. Virol. **168**, 283 (2023). 37904060 10.1007/s00705-023-05906-3 PMC11230039

[40] D. R. Garza, C. A. Suttle, Large double-stranded DNA viruses which cause the lysis of a marine heterotrophic nanoflagellate (Bodo sp) occur in natural marine viral communities. Aquat. Microb. Ecol. **9**, 203–210 (1995).

[41] R. Massana, J. del Campo, C. Dinter, R. Sommaruga, Crash of a population of the marine heterotrophic flagellate Cafeteria roenbergensis by viral infection. Environ. Microbiol. **9**, 2660–2669 (2007). 17922751 10.1111/j.1462-2920.2007.01378.x

[42] P. Hingamp , Exploring nucleo-cytoplasmic large DNA viruses in Tara Oceans microbial metagenomes. ISME J. **7**, 1678–1695 (2013). 23575371 10.1038/ismej.2013.59 PMC3749498

[43] H. Endo , Biogeography of marine giant viruses reveals their interplay with eukaryotes and ecological functions. Nat. Ecol. Evol. **4**, 1639–1649 (2020). 32895519 10.1038/s41559-020-01288-w

[44] M. G. Fischer, M. J. Allen, W. H. Wilson, C. A. Suttle, Giant virus with a remarkable complement of genes infects marine zooplankton. Proc. Natl. Acad. Sci. U.S.A. **107**, 19508–19513 (2010). 20974979 10.1073/pnas.1007615107 PMC2984142

[45] L. J. Reed, H. Muench, A simple method of estimating fifty per cent endpoints. Am. J. Epidemiol. **27**, 493–497 (1938).

[46] H. Li, Minimap2: Pairwise alignment for nucleotide sequences. Bioinformatics **34**, 3094–3100 (2018). 29750242 10.1093/bioinformatics/bty191 PMC6137996

[47] P. Danecek , Twelve years of SAMtools and BCFtools. Gigascience **10**, giab008 (2021). 33590861 10.1093/gigascience/giab008 PMC7931819

[48] J. Besemer, A. Lomsadze, M. Borodovsky, GeneMarkS: A self-training method for prediction of gene starts in microbial genomes. Implications for finding sequence motifs in regulatory regions. Nucleic Acids Res. **29**, 2607–2618 (2001). 11410670 10.1093/nar/29.12.2607 PMC55746

[49] S. F. Altschul, W. Gish, W. Miller, E. W. Myers, D. J. Lipman, Basic local alignment search tool. J. Mol. Biol. **215**, 403–410 (1990). 2231712 10.1016/S0022-2836(05)80360-2

[50] M. Steinegger, J. Söding, MMseqs2 enables sensitive protein sequence searching for the analysis of massive data sets. Nat. Biotechnol. **35**, 1026–1028 (2017). 29035372 10.1038/nbt.3988

[51] T. Nakamura, K. D. Yamada, K. Tomii, K. Katoh, Parallelization of MAFFT for large-scale multiple sequence alignments. Bioinformatics **34**, 2490–2492 (2018). 29506019 10.1093/bioinformatics/bty121 PMC6041967

[52] M. N. Price, P. S. Dehal, A. P. Arkin, FastTree 2–approximately maximum-likelihood trees for large alignments. PLoS One **5**, e9490 (2010). 20224823 10.1371/journal.pone.0009490 PMC2835736

[53] M. Kolmogorov, J. Yuan, Y. Lin, P. A. Pevzner, Assembly of long, error-prone reads using repeat graphs. Nat. Biotechnol. **37**, 540–546 (2019). 30936562 10.1038/s41587-019-0072-8

