# Supplementary material for: Endogenous virophages are active and mitigate giant virus infection in the marine protist Cafeteria burkhardae
Source: Proc Natl Acad Sci U S A. 2024 Mar 6;121(11):e2314606121. doi: 10.1073/pnas.2314606121 (PMC10945749; doi:10.1073/pnas.2314606121)
Supplement: Supplementary file 1 — Appendix 01 (PDF) [file pnas.2314606121.sapp.pdf]

PNAS

Supporting Information for

Endogenous virophages are active and mitigate giant virus infection in the marine protist *Cafeteria burkhardae*.

Anna Koslová, Thomas Hackl, Felix Bade, Alexander Sanchez Kasikovic, Karina Barenhoff, Fiona Schimm, Ulrike Mersdorf, Matthias G. Fischer

Correspondence to Matthias G. Fischer
Email: mfischer@mr.mpg.de

This PDF file includes:

Figures S1 to S7
Tables S1 to S5

Supplemental Figures

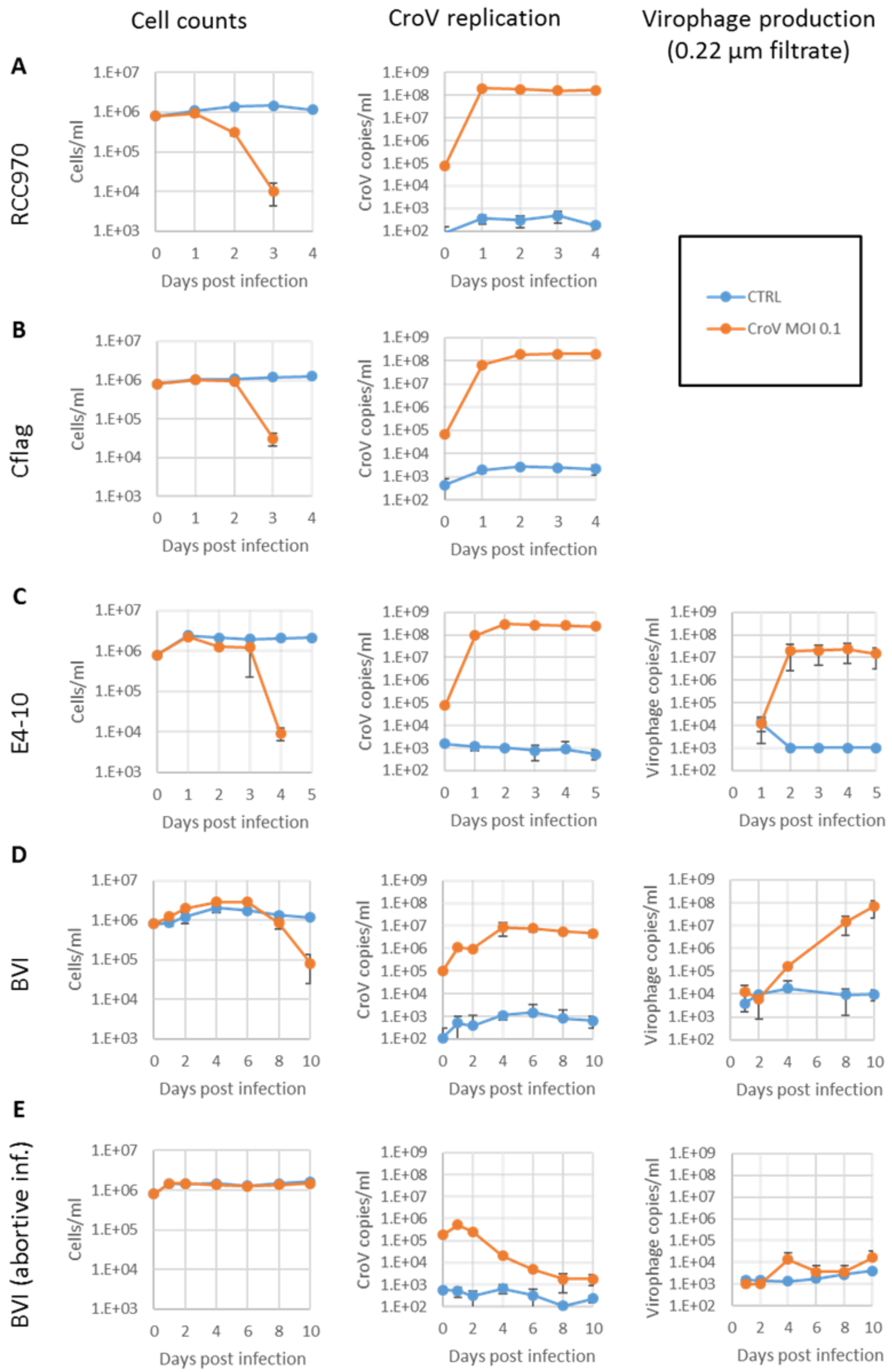

Fig. S1. Dynamics of CroV replication and virophage production during infection of different *C. burkhardae* strains. Four different Cafeteria strains (A-D) were infected with CroV at an MOI of 0.1 and analyzed several days following the infection. Red lines represent infected cultures while blue lines depict uninfected controls. The left column of graphs shows cell densities based on microscopy counts. The middle column of graphs depicts CroV DNA concentrations in the respective cultures as measured by qPCR. The right column of graphs shows the concentration of EMALE04 virophage DNA measured by qPCR in 0.22 µm-filtered culture supernatants. Collection of filtrates started 1 day post infection. CroV infection of host strain BVI did not always result in CroV replication and cell lysis, but sometimes the infection was abortive (E). Plotted values are averages of biological triplicates with error bars representing SD.

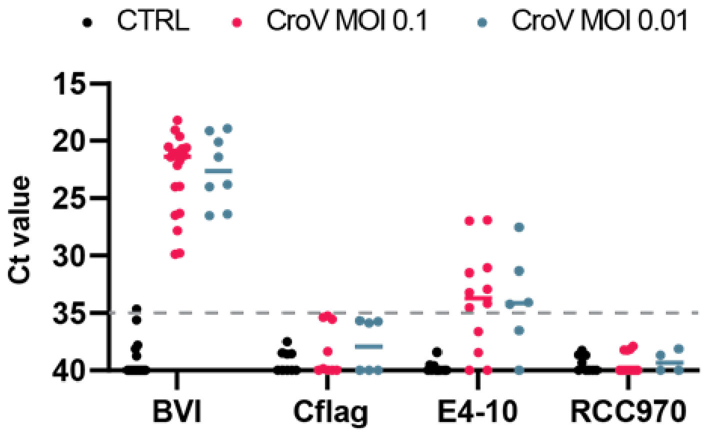

Fig. S2. Measurement of type 4 EMALEs produced during CroV infection of four *C. burkhardae* strains using primers targeting the retroviral integrase gene. The 0.22 µm-filtered lysates were treated with DNase prior to DNA extraction and qPCR analysis with EMALE04-specific *rve-INT* primers. The background threshold is marked with a dashed line.

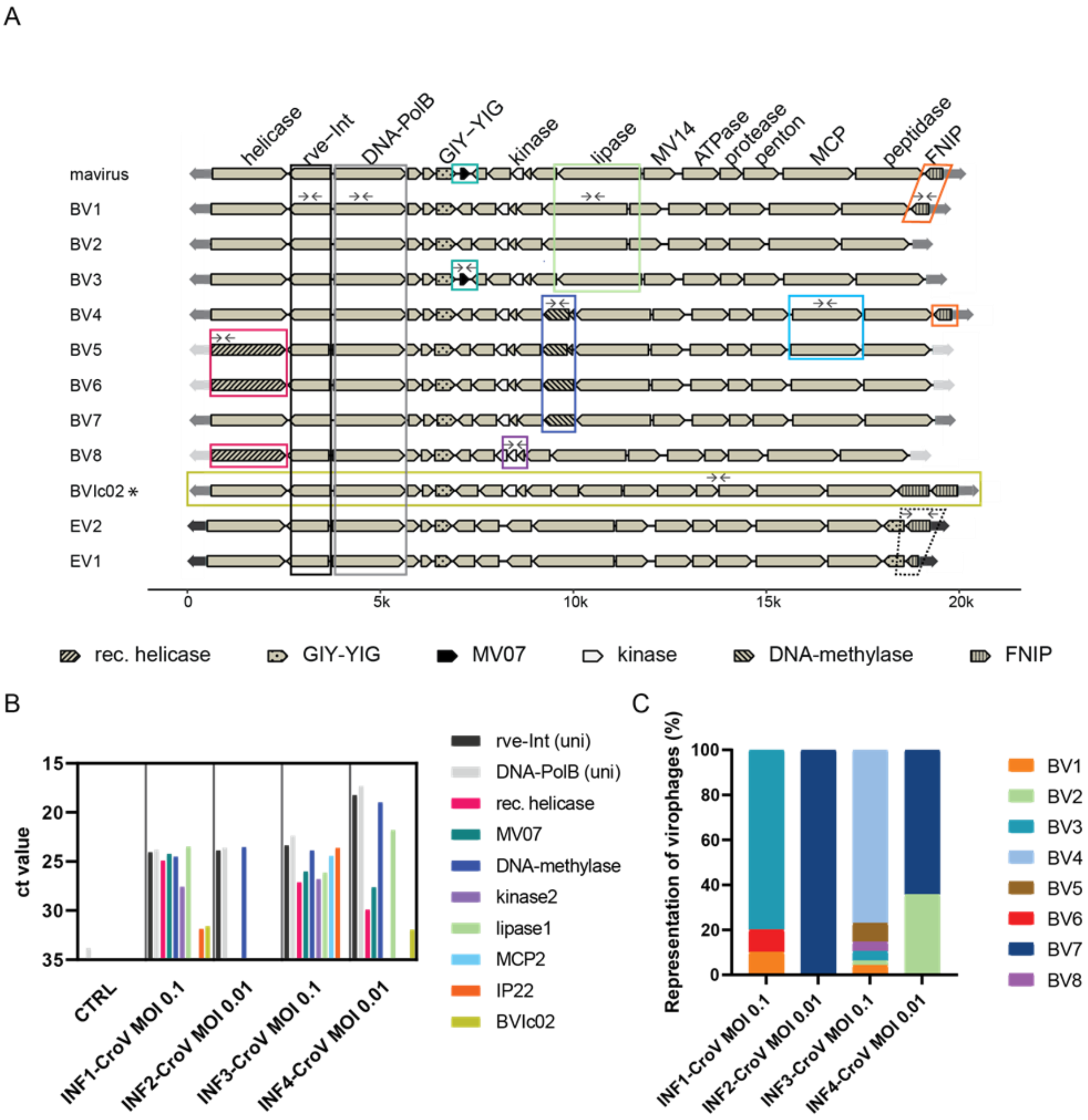

Fig. S3. Different virophages are reactivated during individual CroV infections of *C. burkhardae* strain BVI. A. Genome organization of isolated virophages with depicted positions of qPCR primers targeting either conserved genes (rve-Int, DNA-PolB) or specific genes used to distinguish individual virophages. The colored boxes show which virophages can be detected with indicated primers and the colors also correspond to the legend in B. PCR primers targeting FNIP repeats in EV1/2 are depicted in a dashed box. **B.** Results of qPCR measurements using individual primer pairs targeting the different virophage-specific genes depicted in A. Four individual CroV infections of host strain BVI are shown. **C.** Composition of virophage genomes obtained after cloning using four mixtures of reactivated virophages. Each mixture is the product of an individual CroV infection of host strain BVI.

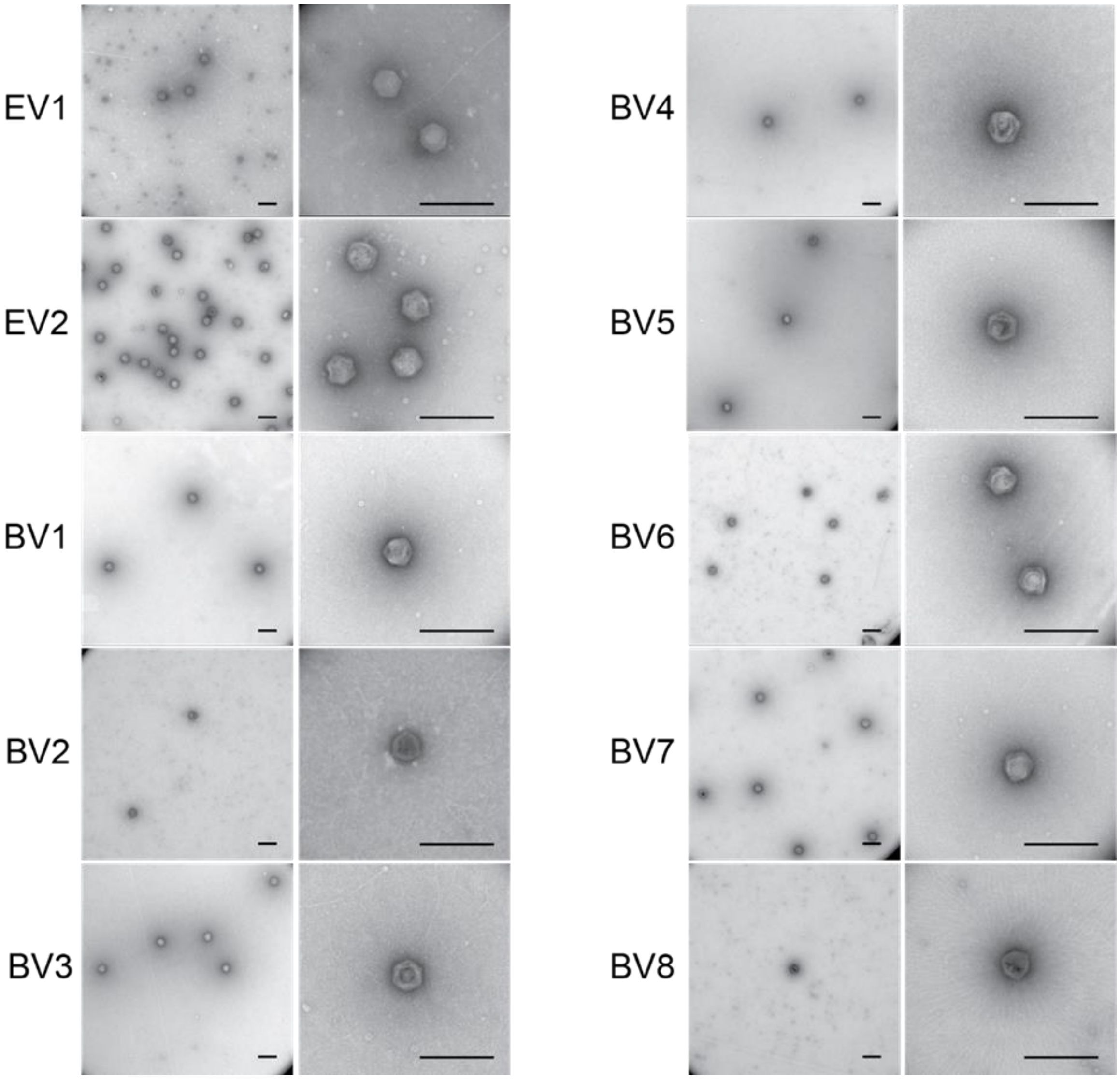

Fig. S4. Negative-stain electron micrographs of individual cloned virophages. Virophages were reactivated from cell clones containing provirophages using CroV infection and the resulting virus particles were concentrated by tangential flow filtration. Scale bars represent 200 µm.

A

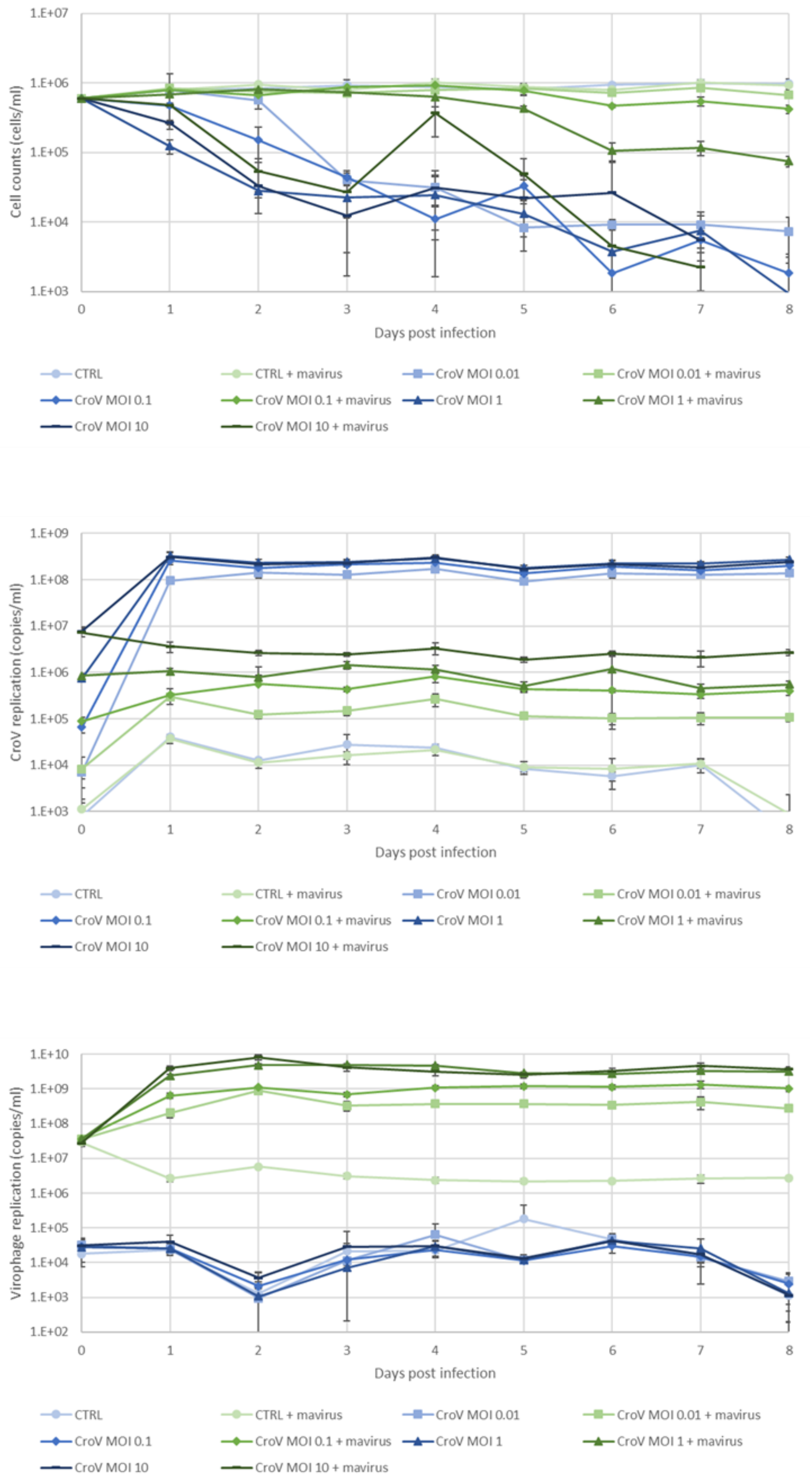
Cell counts (cells/ml)
1.E+07
1.E+06
1.E+05
1.E+04
1.E+03
0 1 2 3 4 5 6 7 8
Days post infection
CTRL
CTRL + mavirus
CroV MOI 0.01
CroV MOI 0.01 + mavirus
CroV MOI 0.1
CroV MOI 0.1 + mavirus
CroV MOI 1
CroV MOI 1 + mavirus
CroV MOI 10
CroV MOI 10 + mavirus
CroV replication (copies/ml)
1.E+09
1.E+08
1.E+07
1.E+06
1.E+05
1.E+04
1.E+03
0 1 2 3 4 5 6 7 8
Days post infection
CTRL
CTRL + mavirus
CroV MOI 0.01
CroV MOI 0.01 + mavirus
CroV MOI 0.1
CroV MOI 0.1 + mavirus
CroV MOI 1
CroV MOI 1 + mavirus
CroV MOI 10
CroV MOI 10 + mavirus
Virophage replication (copies/ml)
1.E+10
1.E+09
1.E+08
1.E+07
1.E+06
1.E+05
1.E+04
1.E+03
1.E+02
0 1 2 3 4 5 6 7 8
Days post infection
CTRL
CTRL + mavirus
CroV MOI 0.01
CroV MOI 0.01 + mavirus
CroV MOI 0.1
CroV MOI 0.1 + mavirus
CroV MOI 1
CroV MOI 1 + mavirus
CroV MOI 10
CroV MOI 10 + mavirus

B

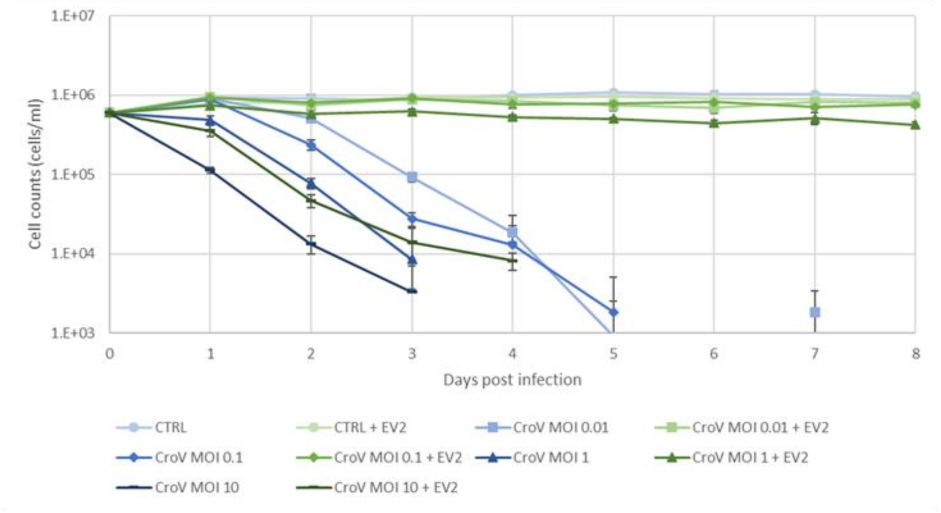
1.E+07
1.E+06
1.E+05
1.E+04
1.E+03
Cell counts (cells/ml)
0
1
2
3
4
5
6
7
8
Days post infection
CTRL
CTRL + EV2
CroV MOI 0.01
CroV MOI 0.01 + EV2
CroV MOI 0.1
CroV MOI 0.1 + EV2
CroV MOI 1
CroV MOI 1 + EV2
CroV MOI 10
CroV MOI 10 + EV2

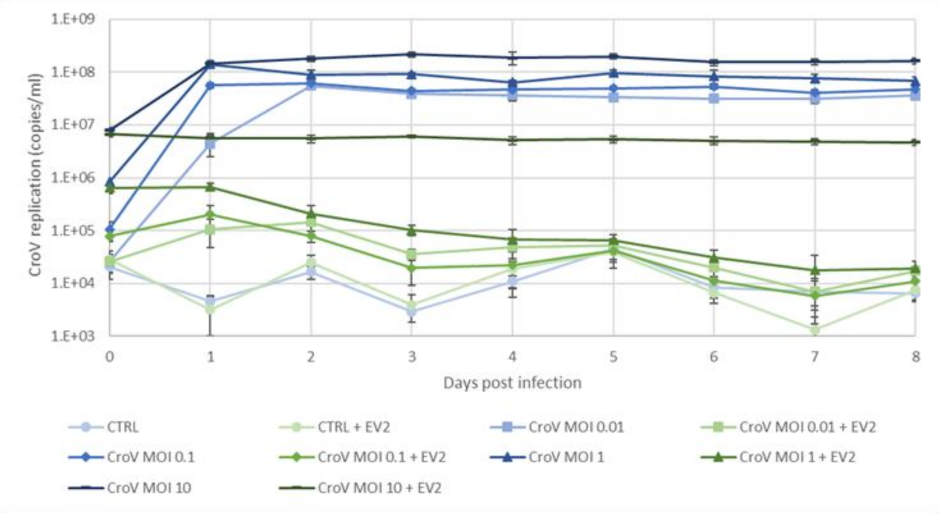
1.E+09
1.E+08
1.E+07
1.E+06
1.E+05
1.E+04
1.E+03
CroV replication (copies/ml)
0
1
2
3
4
5
6
7
8
Days post infection
CTRL
CTRL + EV2
CroV MOI 0.01
CroV MOI 0.01 + EV2
CroV MOI 0.1
CroV MOI 0.1 + EV2
CroV MOI 1
CroV MOI 1 + EV2
CroV MOI 10
CroV MOI 10 + EV2

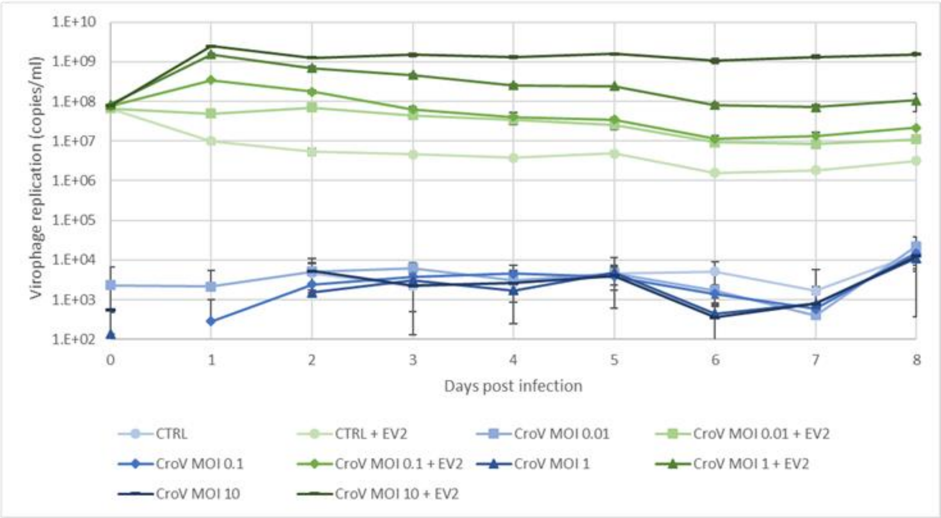
1.E+10
1.E+09
1.E+08
1.E+07
1.E+06
1.E+05
1.E+04
1.E+03
1.E+02
Virophage replication (copies/ml)
0
1
2
3
4
5
6
7
8
Days post infection
CTRL
CTRL + EV2
CroV MOI 0.01
CroV MOI 0.01 + EV2
CroV MOI 0.1
CroV MOI 0.1 + EV2
CroV MOI 1
CroV MOI 1 + EV2
CroV MOI 10
CroV MOI 10 + EV2

C

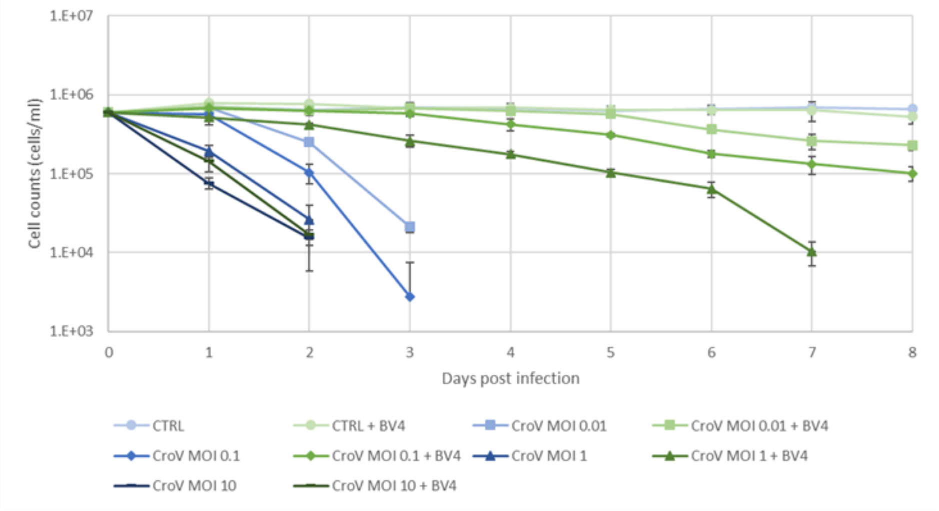
1.E+07
1.E+06
1.E+05
1.E+04
1.E+03
Cell counts (cells/ml)
0 1 2 3 4 5 6 7 8
Days post infection
CTRL
CTRL + BV4
CroV MOI 0.01
CroV MOI 0.01 + BV4
CroV MOI 0.1
CroV MOI 0.1 + BV4
CroV MOI 1
CroV MOI 1 + BV4
CroV MOI 10
CroV MOI 10 + BV4

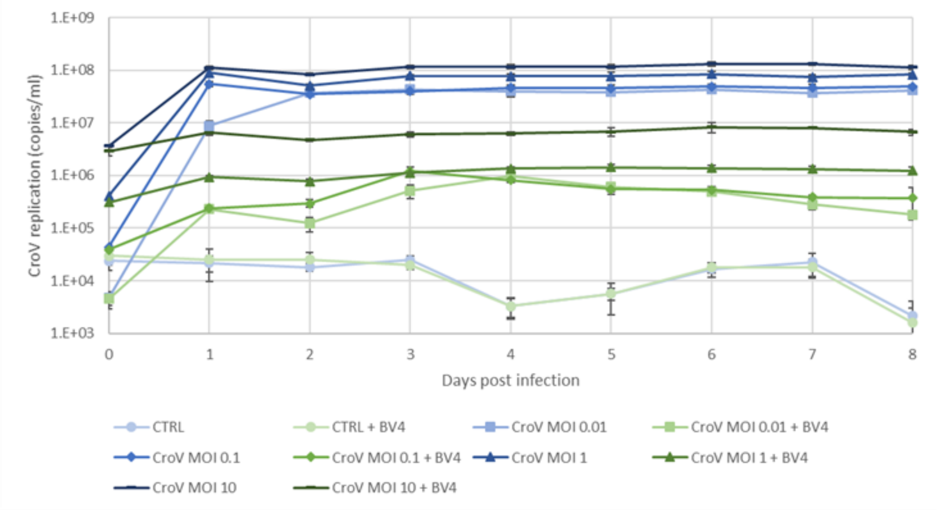
1.E+09
1.E+08
1.E+07
1.E+06
1.E+05
1.E+04
1.E+03
CroV replication (copies/ml)
0 1 2 3 4 5 6 7 8
Days post infection
CTRL
CTRL + BV4
CroV MOI 0.01
CroV MOI 0.01 + BV4
CroV MOI 0.1
CroV MOI 0.1 + BV4
CroV MOI 1
CroV MOI 1 + BV4
CroV MOI 10
CroV MOI 10 + BV4

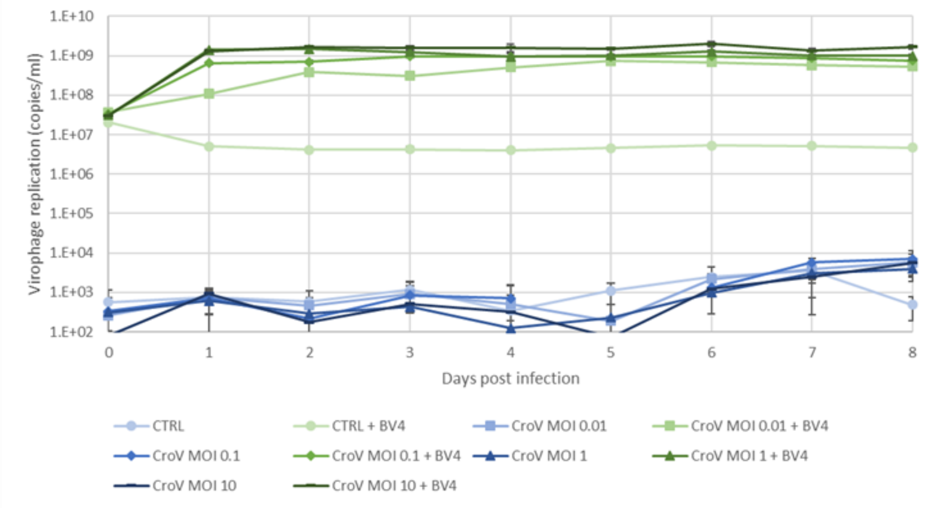
1.E+10
1.E+09
1.E+08
1.E+07
1.E+06
1.E+05
1.E+04
1.E+03
1.E+02
Virophage replication (copies/ml)
0 1 2 3 4 5 6 7 8
Days post infection
CTRL
CTRL + BV4
CroV MOI 0.01
CroV MOI 0.01 + BV4
CroV MOI 0.1
CroV MOI 0.1 + BV4
CroV MOI 1
CroV MOI 1 + BV4
CroV MOI 10
CroV MOI 10 + BV4

D

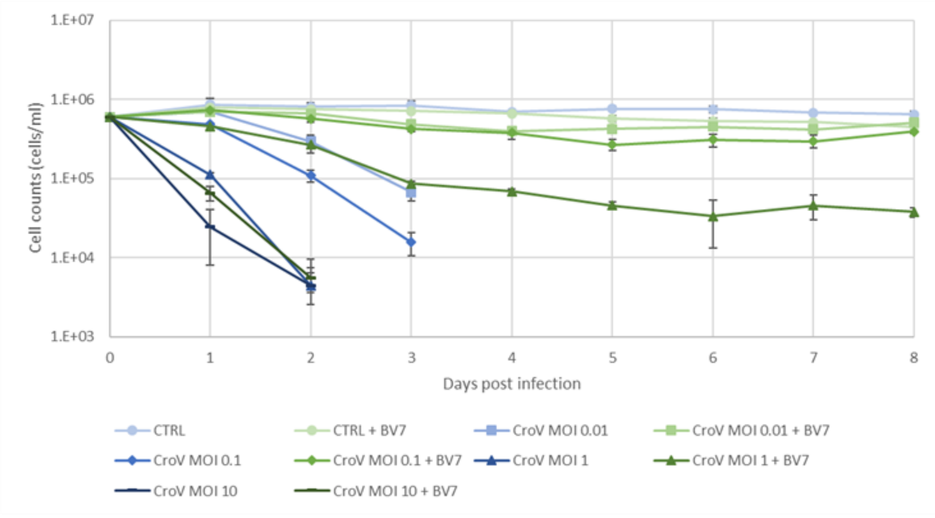
1.E+07
1.E+06
1.E+05
1.E+04
1.E+03
Cell counts (cells/ml)
0 1 2 3 4 5 6 7 8
Days post infection
CTRL
CTRL + BV7
CroV MOI 0.01
CroV MOI 0.01 + BV7
CroV MOI 0.1
CroV MOI 0.1 + BV7
CroV MOI 1
CroV MOI 1 + BV7
CroV MOI 10
CroV MOI 10 + BV7

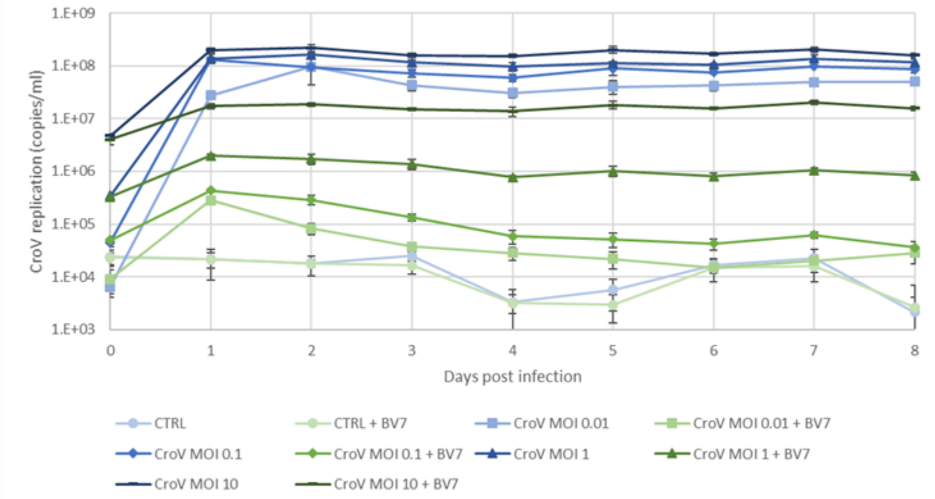
1.E+09
1.E+08
1.E+07
1.E+06
1.E+05
1.E+04
1.E+03
CroV replication (copies/ml)
0 1 2 3 4 5 6 7 8
Days post infection
CTRL
CTRL + BV7
CroV MOI 0.01
CroV MOI 0.01 + BV7
CroV MOI 0.1
CroV MOI 0.1 + BV7
CroV MOI 1
CroV MOI 1 + BV7
CroV MOI 10
CroV MOI 10 + BV7

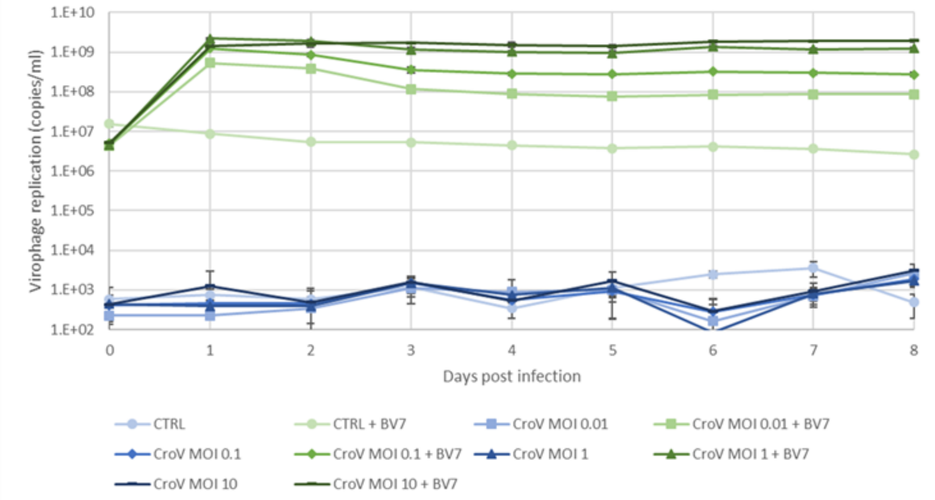
1.E+10
1.E+09
1.E+08
1.E+07
1.E+06
1.E+05
1.E+04
1.E+03
1.E+02
Virophage replication (copies/ml)
0 1 2 3 4 5 6 7 8
Days post infection
CTRL
CTRL + BV7
CroV MOI 0.01
CroV MOI 0.01 + BV7
CroV MOI 0.1
CroV MOI 0.1 + BV7
CroV MOI 1
CroV MOI 1 + BV7
CroV MOI 10
CroV MOI 10 + BV7

E

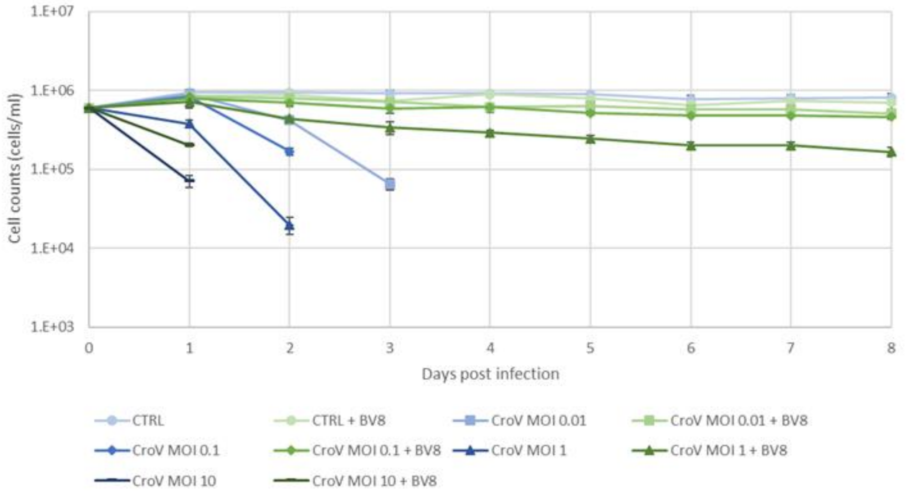
Cell counts (cells/ml)
1.E+07
1.E+06
1.E+05
1.E+04
1.E+03
0 1 2 3 4 5 6 7 8
Days post infection
CTRL
CTRL + BV8
CroV MOI 0.01
CroV MOI 0.01 + BV8
CroV MOI 0.1
CroV MOI 0.1 + BV8
CroV MOI 1
CroV MOI 1 + BV8
CroV MOI 10
CroV MOI 10 + BV8

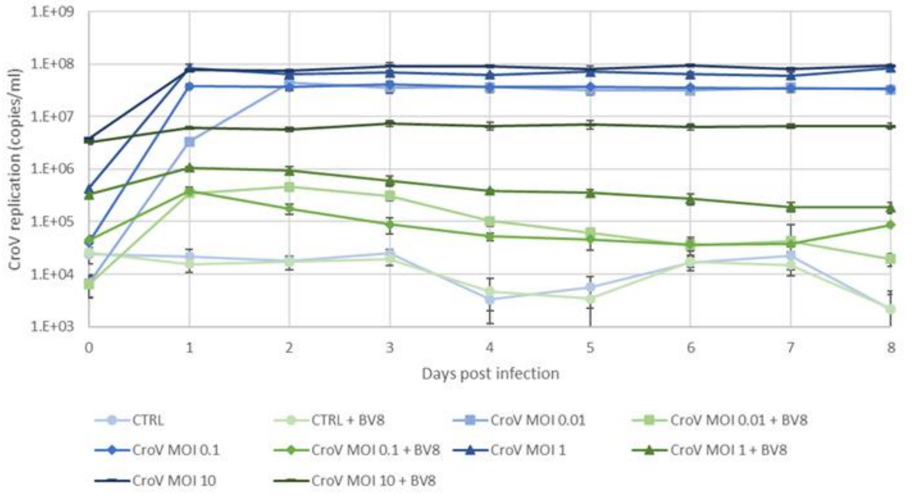
CroV replication (copies/ml)
1.E+09
1.E+08
1.E+07
1.E+06
1.E+05
1.E+04
1.E+03
0 1 2 3 4 5 6 7 8
Days post infection
CTRL
CTRL + BV8
CroV MOI 0.01
CroV MOI 0.01 + BV8
CroV MOI 0.1
CroV MOI 0.1 + BV8
CroV MOI 1
CroV MOI 1 + BV8
CroV MOI 10
CroV MOI 10 + BV8

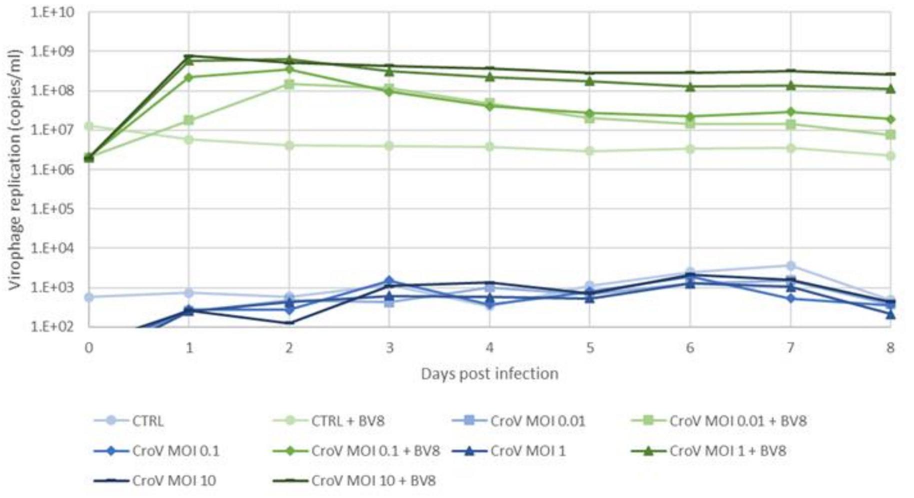
Virophage replication (copies/ml)
1.E+10
1.E+09
1.E+08
1.E+07
1.E+06
1.E+05
1.E+04
1.E+03
1.E+02
0 1 2 3 4 5 6 7 8
Days post infection
CTRL
CTRL + BV8
CroV MOI 0.01
CroV MOI 0.01 + BV8
CroV MOI 0.1
CroV MOI 0.1 + BV8
CroV MOI 1
CroV MOI 1 + BV8
CroV MOI 10
CroV MOI 10 + BV8

Fig. S5. Effect of individual cloned virophages on cell host survival and CroV replication. *Cafeteria burkhardae* strain RCC970 was co-infected with CroV at MOI 0.01-10 and five different cloned virophages at MOI ≈50 (A-E). Cultures were analyzed eight days post infection. Green lines represent co-infected cultures while blue lines depict controls inoculated only with virophages. Top graphs show cell densities based on microscopy counts. Middle graphs depict the amount of CroV gDNA in the culture determined by qPCR. Bottom graphs represent the amount of virophage gDNA in the culture measured by qPCR. Plotted values are an average of biological triplicates with error bars.

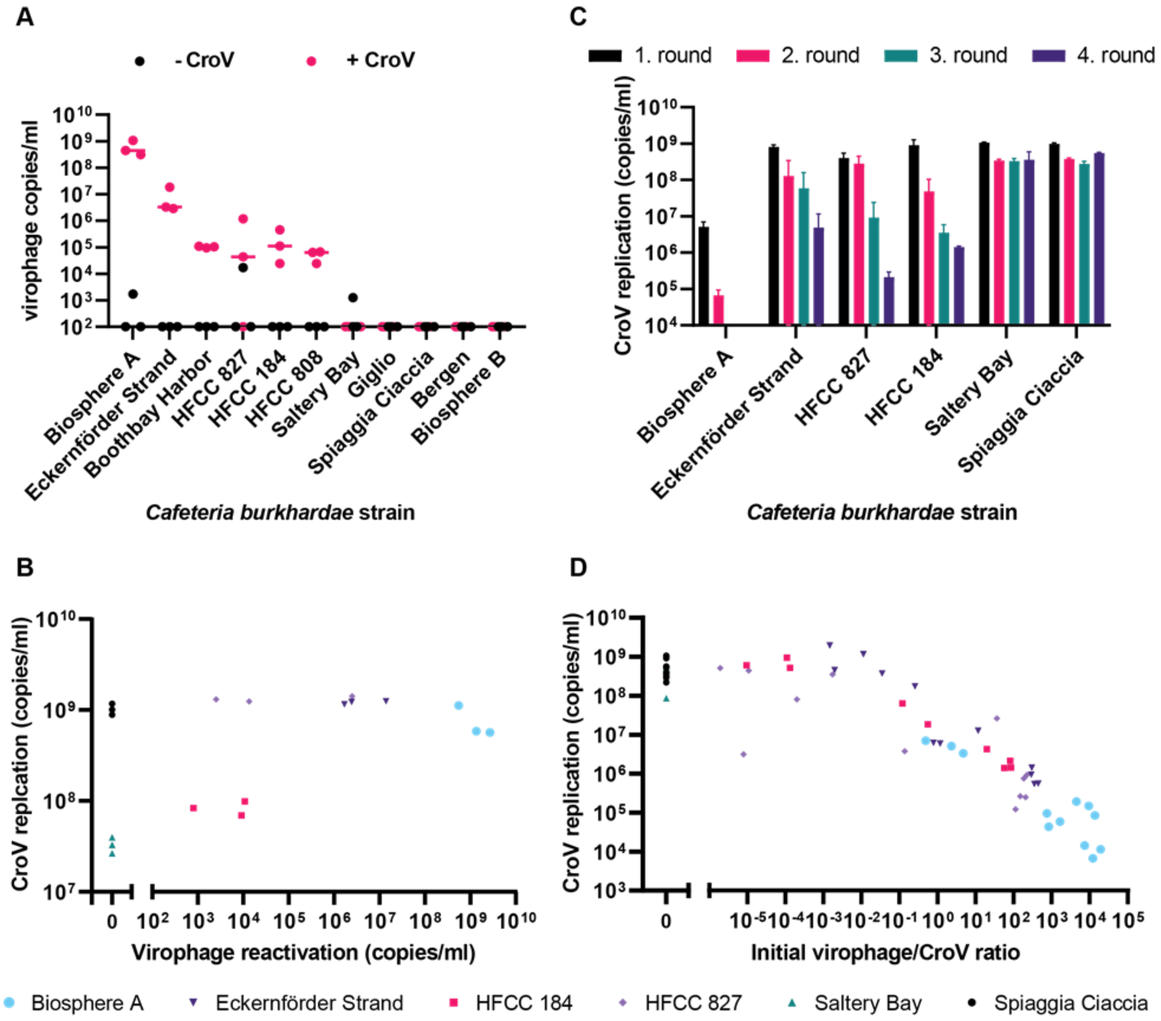

Fig. S6. Virophages reactivated from different *Cafeteria* strains inhibit CroV replication in subsequent rounds of infection. Various *C. burkhardae* strains were infected with CroV at MOI 0.1. After cell lysis, 1.2 µm and 0.22 µm filtrates of cultures were prepared. CroV and virophages in the filtrates were analyzed by qPCR and 1.2 µm filtrates were used for three subsequent rounds of infection of *C. burkhardae* strain RCC970 to analyze the biological activity of reactivated virophages. After each round, the cultures were 1.2 µm filtered and viral copy numbers were monitored. **A.** Quantification of type 4 EMALEs produced during CroV infection of different *C. burkhardae* strains. The 0.22 µm-filtered lysates were treated with DNase prior to DNA release and qPCR analysis with primers specific to the DNA polymerase B gene of EMALE04. **B**. Amount of CroV and virophage DNA copies in 1.2 µm filtrates after CroV infection of individual *C. burhardae* strains was determined using qPCR. **C.** CroV replication in *C. burkhardae* strain RCC970 after infection with 1.2 µm filtrates containing CroV produced from different *C. burkhardae* strains was analyzed by qPCR after each round of infection. **D.** Amount of CroV copies in 1.2 um filtrate is plotted in dependence of virophage/CroV ratio at the time of infection. Assignment to individual *C.*

burkhardae strains is given by origin of the first 1.2 μm filtrate, the following infections were performed in C. *burkhardae* strain RCC970.

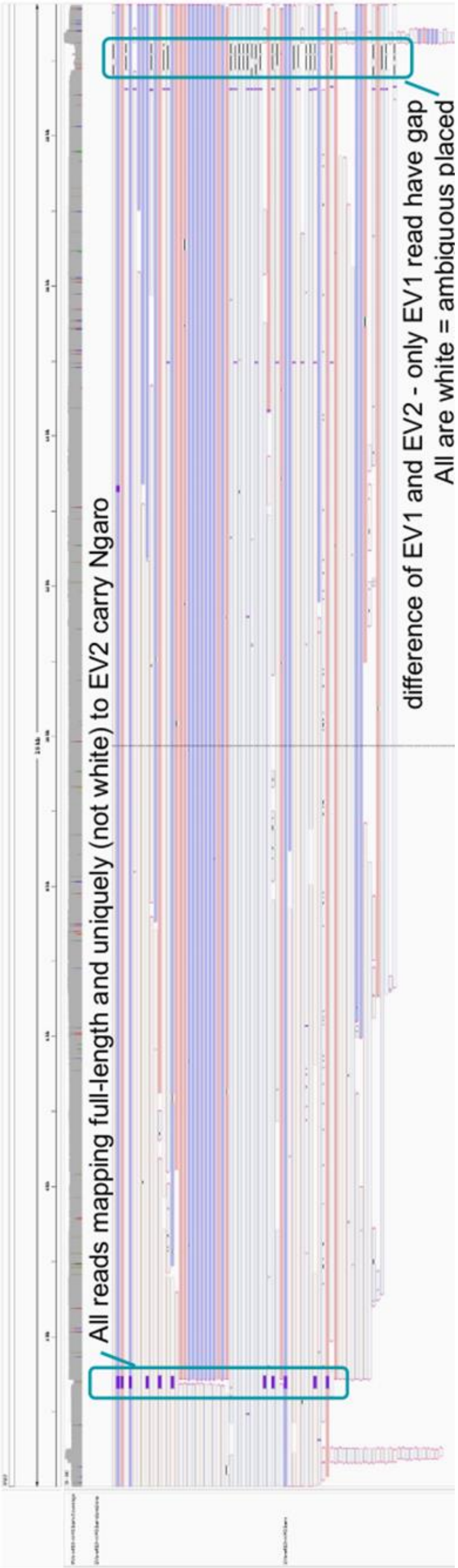
All reads mapping full-length and uniquely (not white) to EV2 carry Ngaro
difference of EV1 and EV2 - only EV1 read have gap
All are white = ambiguous placed

Fig. S7. The endogenous virophage EV2 contains an Ngaro retrotransposon. Nanopore reads longer than 40 kbp from *C. burkhardae* strain E4-10 align in two distinct populations to the genome of EV2. Reads shown in white and with partial deletions at the right-hand end of the genome derive from EV1. All reads without the EV1-specific deletion and aligning close to full-length to EV2 (shown in color) are interrupted close to the left end of the EV2 genome by an Ngaro insertion. Note that the Ngaro insertions themselves are not shown in the figure but were determined by manual analysis of the reads in questions. The exclusive presence of Ngaro-containing EV2-reads in the host genome and Ngaro-free EV2 reads in particles suggests that new Ngaro-free genome copies somehow can be generated during viral reactivation from Ngaro-containing templates.

Supplemental Tables

Table S1. Analysis of DNA contigs from the 0.22 µm-filtered lysate of a CroV-infected E4-10 culture. Post lysis culture supernatant of CroV-infected strain E4-10 was 0.22 µm-filtered and concentrated, followed by Illumina DNA sequencing. Contigs were assembled by Unicycler and analyzed using BLASTn searches (Basic Local Alignment Search Tool) against the nucleotide collection of the NCBI. Only contigs with a sequencing depth greater than 1 are shown.

Contig Number	Length (kbp)	Depth	Best blast hit (Highest NCBI Total Score)	E value	Coverage
1	202.8	1x	Thalassospira indica	0	90 %
4	46.9	2x	Mesorhizobium japonicum	1e-80	1 %
5	45.3 (circular)	13x	Prokaryotic dsDNA virus sp. isolate Unbinned_4811_contig-100_1 (MAG)	0	10 %
6	43.7	160x	Prokaryotic dsDNA virus sp. isolate Unbinned_4811_contig-100_1 (MAG)	5e-115	1 %
8	41.5	1x	Pseudomonas sp. Leaf58 chromosome	6e-05	0 %
9	40.5 (circular)	19x	Microbacterium chocolatum strain SIT 101	0	3 %
10	39.4	20x	Prokaryotic dsDNA virus sp. isolate Unbinned_4811_contig-100_1 (MAG)	0	28 %
11	33.7	1x	Maribellus comscasis strain WC007 chromosome	4e-06	0 %
12	26	1x	Rhizobiales bacterium NRL2 chromosome (MAG)	0	36 %
**16**	**16.3**	**254x**	**Cafeteriavirus-dependent mavirus isolate endo_mavirus**	**0**	**47 %**

Table S2. Analysis of clones established after 4 different inoculations of RCC970 strain of *C. burkhardae* with BVI reactivated virophages. Comparison of the number of provirophages per genome detected by qPCR analysis of mixed populations and the number of clones containing integrated virophages.

	**Provirophages per genome (qPCR - ΔΔCt method)**	**Analyzed clones**	**Clones containing provirophages**	**Provirophage positive clones (%)**
**INF1-CroV MOI 0.1**	0.07	102	10	9.8
**INF2-CroV MOI 0.01**	0.2	82	16	19.5
**INF3-CroV MOI 0.1**	0.3	137	48	35
**INF4-CroV MOI 0.01**	0.22	96	14	14.6

Table S3. Information on *Cafeteria* sp. strains used in this study. All strains are classified as *C. burkhardae*, except for the two marked with an asterisk that likely belong to a different species.

***Cafeteria* strain**	**Location**	**Coordinates**	**Year**	**Isolated by**
BVI	Caribbean Sea, British Virgin Islands	18.4258N 64.6175W	2012	I. Schlichting, M.G. Fischer
Cflag	North Atlantic Ocean, Woods Hole, MA	41.5261N 70.6741W	1986	D. Caron
E4-10P	North Pacific, 5 km west of Yaquina Bay, Oregon	44.6239N 124.075W	1989	J. Gonzales, E. & B. Sherr, A. Chan
RCC970-E[3] (now RCC4623)	South Pacific Ocean, 2200 km off the coast of Chile	30.7833S 95.4333W	2004	L. Garczarek, D. Marie
Biosphere 2, clone A	Biosphere 2 Artificial Ocean, Oracle, USA	32.5786N 110.8503W	2012	M.G. Fischer
Eckernförder Strand	SW Baltic Sea, Beach in Eckernförde, Germany	54.4710N 9.8407E	2020	M. Musillo
Boothbay Harbor	NW Atlantic, Coastal shoreline near Boothbay Harbor, USA	43.8142N 69.5939W	2019	M.G. Fischer
HFCC827	SW Pacific, East of Vanuatu	15.2169S 173.5186E	2012	H. Arndt
HFCC184	Central Atlantic, SW of Cape Verde	10.4636N 31.0272W	2014	H. Arndt
HFCC808	West Pacific, SE of Guam	10.5717N	2012	H. Arndt

		148.8169E		
HFCC197	West Atlantic, North of Puerto Rico	19.7669N<br>66.8167W	2015	H. Arndt
HFCC168	Central Atlantic, SW of Cape Verde	10.4186N<br>31.0769W	2014	H. Arndt
Saltery Bay clone 1	NE Pacific, Beach in Mermaid Cove, Saltery Bay Provincial Park, Canada	49.7794N<br>124.1914W	2013	M.G. Fischer
IOW23	Baltic Sea coastal monitoring station near Heiligendamm (sea bridge)	54.146N 11.843E	2003	A.P. Mylnikov
Giglio 5 clone2	Mediterranean Sea, Spiaggia delle Cannelle, Giglio Island, Italy	42.3506N<br>10.9206E	2012	1994M.G. Fischer
RCC1071 mono 1	SE Pacific	31.8167S<br>91.4667W	2004	L. Garczarek, D. Marie
RCC1068 G2-B7-G6	SE Pacific, NW of Hanga Roa	25.983S<br>113.983W	2004	D. Vaulot, D. Marie
RCC257 clone 1	Central Atlantic, NW of Cape Verde	21.03N<br>31.13W	1991	F. Partensky
RCC1079	SE Pacific, SE of Hanga Roa	27.767S<br>107.283W	2004	L. Garczarek, D. Marie
Spiaggia Ciaccia	Mediterranean Sea, Beach in La Ciaccia, Sardegna, Italy	40.9192N<br>8.7825E	2012	M.G. Fischer

CCAP 1900/2 mono5 B	White Sea, North of Louhi	66.2700N<br>33.0708E	1986	A. P. Mylnikov
Bergen 2 clone 2	North Sea, Bergen Harbor, Norway	60.3947N<br>5.3247E	2014	I. Schlichting, M.G. Fischer
Biosphere 2, clone B	Biosphere 2 Artificial Ocean, Oracle, USA	32.5786N<br>110.8503W	2012	M.G. Fischer
RCC22 clone 1	Central Pacific, North of French Polynesia	11.50S<br>150.00W	1994	D. Vaulot
Plymouth clone 1*	English Channel, Beach near West Hoe Park	50.3639N<br>4.1461W	2016	M.G. Fischer
Rock Crack G4 L	English Channel, Tidal Pool near Porthchapel, UK	50.0375N<br>5.6617W	2016	M.G. Fischer
Porth Chapel mono 1*	English Channel, Porthchapel Beach, UK	50.0386N<br>5.6569W	2016	M.G. Fischer

Table S4. Links between reactivated virophage genomes and endogenous elements in the nuclear genomes of *Cafeteria* strains E4-10 and BVI. Sequences of isolated virophages were searched by BLAST against the draft genome assembly of host strain E4-10 or BVI. EMALEs with similarity higher than 97 percent and containing unique features of specific virophage were considered as matches.

Virus	Most similar EMALE	Ngaro	Nucleotide Identity (%)	Completeness	Unique feature
EV1	E4-10-contig3803	No	99	full EMALE	FNIP-EV1
EV2	E4-10-contig95	Yes	99 (without Ngaro)	full EMALE	FNIP-EV2
BV1	not found				Lipase1+FNIP
BV2	BVI-contig25	Yes	98 (without Ngaro)	full EMALE	Lipase1
BV3	BVI-contig88	No	99	full EMALE	Lipase1+MV07
BV4	not found				Helicase1+DNA-methylase+MCP2
BV5	BVI-contig39A	No	100	full EMALE	Helicase2+DNA-methylase+MCP2
BV6	not found				Helicase2+DNA-methylase+MCP1
BV7	BVI-contig29	No	100	full EMALE	Helicase1+DNA-methylase+MCP1
BV8	BVI-contig50	Yes	97 (without Ngaro)		Helicase2 +kinase2

Table S5. List of primers used for qPCR and PCR.

Primer	DNA Sequence (5'→ 3')	Target
Int-2-fw	GGGTTGATTTACTGCCCGAC	MV02, rve-Int of EMALE04
Int-2-rv	TTCACAAGCATCACCCTTACA	MV02, rve-Int of EMALE04
MaV-Pol-fw	GCGATGAATTGTTTATGGGGTGC	MV03, PolB of EMALE04
MaV-Pol-2-rv	AGTTTGTATCTTCCTTTTGCTGTT	MV03, PolB of EMALE04
CroV-qPCR-9	CTAAATTGGCCAGGTCTGGGTCTT	crov283 D11-like tissue factor (TF)
CroV-qPCR-10	CGTGGTAGAGTGGGTGAGAATGAA	crov283 D11-like TF
Spezl-qPCR-5	TAGTGGTGCTCTGGCTAATGGCTT	MV18 (MCP)
Spezl-qPCR-6	TTAATCCCAGATCGGAAGGACGGA	MV18 (MCP)
Cr_E4-10-AspRS-fw	CATGGAGATGACCTTCAACG	AspRS
Cr_E4-10-AspRS-rv	GAAGTCCTCGTGGGGGTACT	AspRS
EMALE-BVIc55-434-fw	TATTCAACGGGCCCATCACG	BV-Rec. helicase
EMALE-BVIc55-545-rv	TGTGGTAAGATTTGTGGAGCCA	BV-Rec. helicase
EMALE-BVIc02-6571-fw	TGTACCAACATCAGTAACGCCT	BVIc02
EMALE-BVIc02-6747-rv	AAAGGAGCCAATAAAGAAAACGT	BVIc02
EMALE-BVIc16-2858-fw	CGTAAAGCCTCACCCATACG	MV07
EMALE-BVIc16-2983-rv	ACGTTGGTCAGTGGTCAATCT	MV07
EMALE-BVIc16-15290-fw	GCGGTTGATTAAATCTTTTGTCATCA	BV-FNIP/FG-repeats
EMALE-BVIc16-15460-rv	AATGCGTGGTGTTTGGAAAA	BV-FNIP/FG-repeats
EMALE-BVIc74-8957-fw	AGGAGATGATGACGATGATGACG	BV-Lipase1
EMALE-BVIc74-9232-rv	GTTTGTGCCCTTGATCGTGT	BV-Lipase1
EMALE-BVIc122-5903-fw	AACTCATCCGCGGGTAAATTT	DNA-methylase
EMALE-BVIc122-6036-rv	GCGGATTCTGTTAGTGGATGT	DNA-methylase
EMALE6-MCP-fw	ATGATGTGGCACTTCCCGATG	BV-MCP2
EMALE6-MCP-rv	TATATGGGGCTCGATCATTTATACATG	BV-MCP2

EMALE-BVIc120-4430-fw	GGTACTCTCTACCCTCTATAATAAAA	Kinase2
EMALE-BVIc120-4499-rv	TTAAGAAATATTAGAGAGTGCAGTGA	Kinase2
EMALE-E410P-FG-fw	CGGGACAATCAGGAGAACAAATTTT	EV-FNIP/FG-repeats
EMALE-E410P-FG-rv	ACTGACTTTGGTTATGCTTTATCC	EV-FNIP/FG-repeats
